# Leveraging *Lactobacillus plantarum* probiotics to mitigate diarrhea and Salmonella infections in broiler chickens

**DOI:** 10.1186/s13568-024-01792-3

**Published:** 2024-12-18

**Authors:** Seyed Mehrdad Mirsalami, Mahsa Mirsalami

**Affiliations:** 1 Department of Chemical Engineering, Faculty of Engineering, Islamic Azad University Central Tehran Branch, Tehran, Iran; 2 Faculty of Engineering and Technical Sciences, Qazvin Islamic Azad University, Qazvin, Iran

**Keywords:** Anaerobic bacteria, *Lactobacillus plantarum*, Probiotics, Salmonella, Diarrhea, Broiler chickens

## Abstract

Anaerobic bacteria, such as *Lactobacillus plantarum* (LP), are known to play a significant role in maintaining gut health and protecting against enteric pathogens in animals. The present study aimed to develop a safe, affordable, and eco-friendly method for producing LP-based probiotics and evaluate their efficacy in mitigating Salmonella-induced diarrhea in broiler chickens. The study employed three different culture media (MRS, TSB, and Baird Parker) to grow LP, which was then dried using a spray-drying technique to produce a stable probiotic formulation. When administered to broiler chickens, the LP probiotic derived from the MRS medium significantly improved body weight gain (4.147-fold increase over 4 weeks) compared to the other two culture conditions. Importantly, the LP probiotic treatment could substantially reduce the diarrhea index in broilers, with up to an 86.45% improvement in Salmonella-induced enteric infections. The beneficial effects were attributed to the ability of LP to modulate the gut microbiome, enhance the integrity of the intestinal mucosa, and mitigate the pathogenic effects of Salmonella. These findings demonstrate the potential of anaerobic *Lactobacillus plantarum* as a safe and effective probiotic intervention for controlling enteric diseases and improving production outcomes in poultry farming. The developed method provides a sustainable approach to harness the beneficial properties of this anaerobic bacterium for animal health and welfare.

**Graphical abstract:**

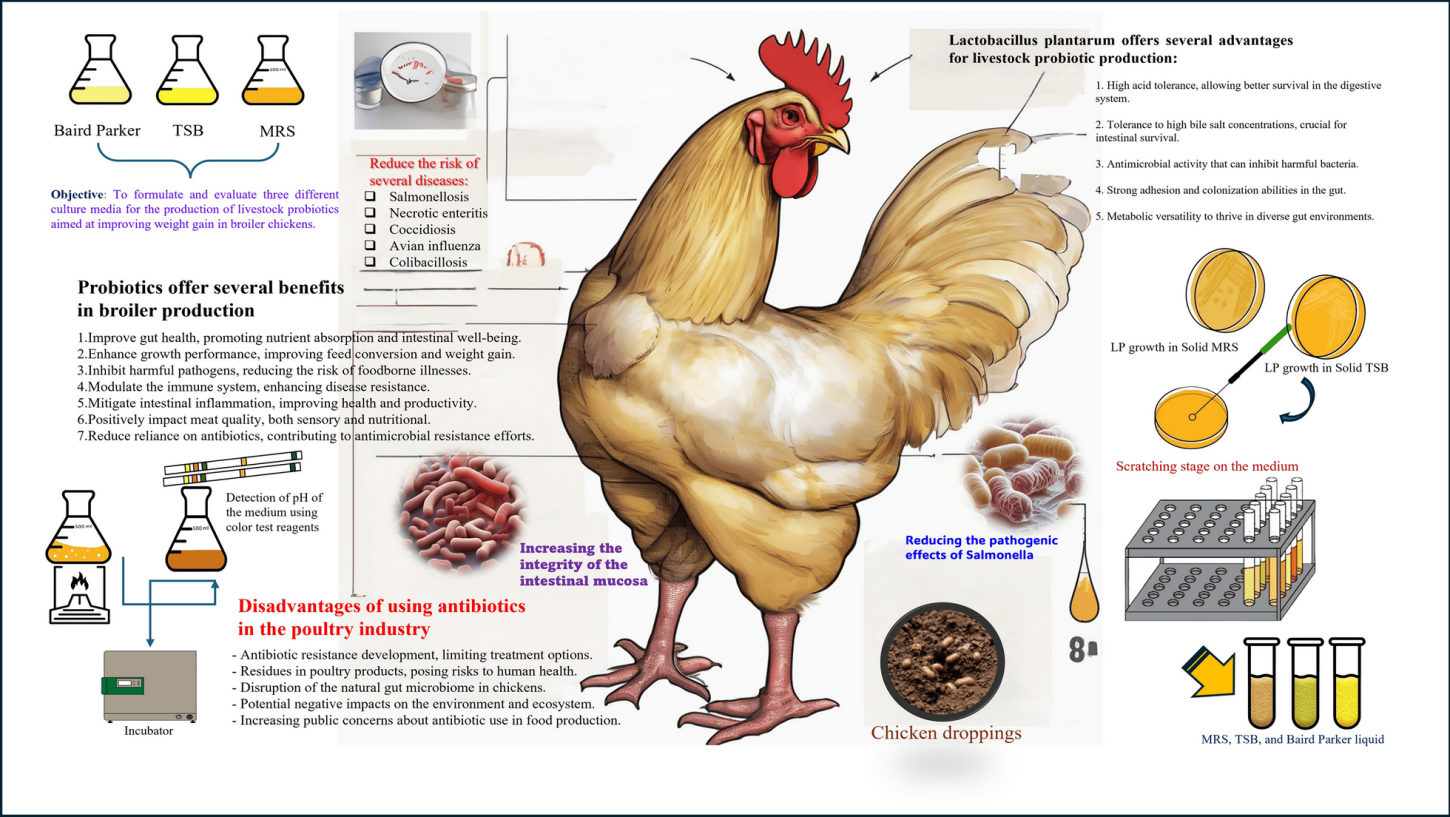

**Supplementary Information:**

The online version contains supplementary material available at 10.1186/s13568-024-01792-3.

## Introduction

Probiotics, as defined by the WHO and FAO are "living microorganisms that affect the parity of useful and detrimental bacteria in the gut and promote this balance in favor of increasing populations of beneficial bacteria change (Archacka et al. 2020). When given in sufficient quantities, they bring health benefits to the host." Live microorganisms can have useful outcomes such as anti-inflammatory, anti-pathogenic and reduce lactose intolerance on the host if consumed in sufficient quantities (Sebouai et al. 2024). In recent decades, due to a global trend to restrict the use of antibiotics in animal feed, and the function of probiotics in increasing the weight and appetite of animals to eat more fodder was introduced as a potential alternative to antibiotics in maintaining the health of animal feed and reducing antimicrobial resistance (de Marins et al. 2022). Consumption of industrial food has increased dramatically in recent years, with the food and dairy industries using harmful additives in their composition (Alomayri et al. 2014). However, more and more consumers are concerned about the use of these synthetic additives in their regular diets. Consequently, the need for indigenous and green foods free of chemical preservatives is significantly greater than past. The growing inclination towards minimally processed meats has intensified the pursuit for Indigenous and non-chemical maintainers, hence probiotic combinations such as *Lactobacillus* meet these demands (Oloruntola et al. 2024).

*Lactobacilli* are generally fermentative microorganisms that can measure up to ten microns in size. The majority of their metabolic byproducts consist of lactic acid (LAB), and they possess beneficial probiotic characteristics. Consequently, *Lactobacillus* is recognized as a significant probiotic bacterium with particular capabilities in the production of food-related animal products (Sun et al. 2022). Among 150 distinct species of lactic acid bacteria (LAB), only two LP and *β-langum* demonstrate superior resilience to both temperature variations and laboratory environments. LP is one of the most abundant kinds of the genus-favorable bacteria and is extensively used in dairy-meat industry-relevant technologies (Cao et al. 2019). This strain is a significant industrial microorganism which can be extracted from fermented foods of broccoli, wine, olives, wiener and dairy products such as cheese, yogurt, and buttermilk and also from the animal enteric, feces and live mouth (Wirunpan et al. 2016). By supporting the growth of LP, the nutrient culture medium enhances its efficacy in modulating the intestinal microbiome of broiler chickens. This probiotic is known for its ability to adhere to the intestinal lining, outcompeting harmful pathogens and contributing to a balanced microbiota. Furthermore, the fermentation products produced by LP, such as lactic acid, can lower the pH of the gut environment, inhibiting the growth of pathogenic bacteria and improving overall gut health. This dual action not only promotes the well-being of the chickens but also leads to improved meat quality characteristics, making the use of a nutrient culture medium an essential component in probiotic applications for poultry production (de Marins et al. 2022). Over the years, it has been proven the desired results not only on curing the inductive defensive system but also in improving prevalent infections, inclusive of respiratory infection, genitourinary, and gastrointestinal (Paul et al. 2017).

The intestinal microbiome consists of a diverse community of microorganisms that inhabit the gastrointestinal tract of poultry. This complex ecosystem plays a crucial role in digestion, nutrient absorption, and overall health. A balanced microbiome can enhance the immune response, reduce the incidence of gastrointestinal diseases, and improve feed efficiency. Moreover, emerging research suggests that the microbiome significantly influences meat quality characteristics, including flavor, tenderness, and nutritional composition. By modulating the gut microbiota through probiotics, it is possible to enhance these attributes, ultimately leading to better product quality and consumer acceptance.

Therefore, it has lately been considered as an impressive strategy to improve diarrhea in chickens and use it in fishponds to become more voluminous in the shortest possible time. Probiotics can affect the bird's defense mechanism and increase the production of antibodies, especially (IGM and IGG), and probiotics also have the potential to control fat and protein levels in animals (Guerrini et al. 2024), which increases the lifespan of living things. Chicken meat has been generally related to dietary protein Salmonella infections. Salmonellosis has been a major cause of hospitalization in the world over the past decade. Amongst the *S. Enterititis*, *S. enterica* serovars has been identified as the main factor of food poisoning universal and the bacteriophage-type (PT-4) one of the most malignant (da Silva Sabo et al. 2014). Recent research has shown that giving excessive amounts of probiotics to aquatic and poultry animals reduces their beneficial effects, as it may cause competition between hosts and bacteria over food (Sebouai et al. 2024). Bacteriocin produced by bacteria may also kill the bacterium itself in high concentrations. The sooner any germs enter the gastrointestinal tract, the more likely they are to settle in the gastrointestinal tract. For this reason, it is recommended that probiotics be used from the beginning of life and prevent the establishment of harmful bacteria in the digestive system (Guardiola et al. 2021).

The investigation into the role of probiotics, particularly *Lactobacillus plantarum*, in enhancing meat quality and broiler chicken health is critical due to existing gaps in the current literature. While numerous studies have explored the effects of probiotics on gut health, there is a lack of consensus regarding their impact on specific meat quality characteristics such as taste, texture, and nutritional value (Suez et al. 2018). Some research suggests positive outcomes, while others report minimal or inconsistent effects, highlighting the need for further investigation. Additionally, the reliance on antibiotics in poultry production poses significant health risks, including antibiotic resistance, which underscores the urgency of identifying effective alternatives like probiotics (Plaza-Diaz et al. 2019). Optimizing the intestinal microbiome through the application of probiotics offers several benefits for the poultry industry, including improved animal health, enhanced meat quality, and increased food safety. Healthier chickens can lead to reduced mortality rates and lower veterinary costs, contributing to economic viability for producers (Skoufou et al. 2024). Environmentally, utilizing probiotics can decrease the need for antibiotics, thereby promoting sustainable farming practices and reducing the ecological footprint of poultry production. By addressing these gaps and exploring the multifaceted benefits of probiotics, this research aims to contribute valuable insights that can enhance both industry practices and consumer health.

The primary objective of this study is to evaluate the impact of *Lactobacillus plantarum* on the intestinal microbiome of broiler chickens and its effects on meat quality traits, including taste, texture, and nutritional value. Additionally, the research aims to assess the probiotic's role in enhancing food safety by mitigating pathogenic microorganisms, notably Salmonella, and improving the overall health of the chickens. By examining the probiotic's ability to modulate the gut microbiome and strengthen intestinal integrity, this study seeks to provide evidence-based recommendations for the effective use of *Lactobacillus plantarum* in poultry farming, ultimately benefiting producers, consumers, and public health.

To achieve this, various culture media such as MRS, TSB, and Baird Parker were utilized to optimize the growth of *Lactobacillus plantarum*. These media provide essential nutrients that support the robust proliferation of the probiotic, ensuring that it reaches effective levels for application in poultry diets. Furthermore, the probiotic was dried using an innovative spray drying method, resulting in a stable formula that can be easily integrated into feed. This method not only preserves the viability of the probiotic but also enhances its practicality for use in commercial poultry farming.

## Materials and methods

### Materials

In this study, LP-BL0111 was considered as a model probiotic strain. The unadulterated strain was acquired from PROBIOTIC SUPPLIERS Ltd. (Toronto, Ontario, Canada) in powdered form and kept at −22 °C. The main components of the content of the culture medium include D(t)-glucose, peptone from casein, meat extract, glycine and sodium pyruvate. The raw sequence data generated in this study have been deposited in the NCBI Sequence Read Archive (SRA) under the BioProject accession number PRJNA1168573. The specific SRA accession number for the data can be found at SRP528572.

### Method

The desired strain of L. plantarum, which is both aerobic and anaerobic, has been considered for the experiment. As a heterogeneous species, it is considered to be resistant to acid and a safe microorganism and is closely related to *Lactobacillus helveticus*, *L. acidophilus*, and, more recently, *Lactobacillus fabiformes*. This proportion was found when 99% of their rRNAs reported the same subsequence, indicating a high interaction of genotype and observable characteristics similarity between species (Voo et al. 2022). In the first experiment, three types of culture medium were prepared in powder form and their pH was kept constant in the same neutral range, and according to Table 1, the required amounts of culture medium were dissolved in 200 ml of pure water. To prevent the entry of impurities, we close the lids of the containers and put them on the flame, and 5 min is provided for complete dissolution of the powder. The medium was then placed in an autoclave (121 °C) for 15 min. After this period has elapsed, wait for the culture medium to cool slightly, and pour them on the plates and allow the prepared culture medium to close on the plates. In the second step, we prepared the two medium MRS (de Man, Rogosa, and Sharpe) and TSB (Tryptic Soy Broth) again and added 2 (gr) of agar and performed the previous steps again.

**Table 1:** Components of MRS, TSB, Baird parker medium

Type of medium	Components	Amount (g/L)	pH	Amount of preparation (g/L)
MRS	Pepton from casein	10.1	6.5 ± 0.1	55.15
	Meat extract	8.9		
	Yeast extract	4.72		
	D(t)– Glucose	20.63		
	Di- potassium hydrogen phosphate	2.23		
	Tween 80	1.74		
	Di-Amoniumhydrogen citrate	2.39		
	Sodiym chloride	5.56		
	Sodium acetate	5.12		
	Magnesium sulfate	2.08		
	Manganese sulfate	0.04		
TSB	Peptic digest of soybean meal	3.44	7.3 ± 0.2	30
	Sodium chloride	5.9		
	Di-potassium hy drogen phosphate	2.58		
	Dextrose	2.5		
Baird parker	Casein peptone	10	6.8 ± 0.2	60
	Yeat extract	1.08		
	Beef extract	5.67		
	Lithium chloride	5.02		
	Sodium pyruvate	10.1		
	Glycine	12.6		
	Agar	17.4		

### PMs assessment

In this laboratory-scale study, the Phenotype MicroArrays (PMs) benchmark was executed by instrumentation related to this method (Biolog, USA) and PM1-2 sheet. The PM1-2 is a particular template or “identification of a unique metabolic pattern” from separate trial passivity performed inside sealable 96-well plates by Biology's powerful carbon source utilization technology. L. plantarum was uniformly implanted at 36.5 °C on pre-sample agar medium and afterward was washed in IF-0 fluid to attain the goal cell compactness (aligned with 80–85% transition). The initial sheet (plate) was immunized via 150 μL of the cell suspension and it was subjected in optimal conditions of 36–37 °C for 72 h. Within the growth of microorganisms, triphenyl color existence in the culture medium decreased in those wells where absorbable C-source was included.

The color changes of the culture medium during the reaction time are clearly recognizable so that the initial colorless substrate gradually turns purple, which was measured by special platform (OmniLog) during the term the cultivation.

### Cultivation of bacteria on bioreactor

The greater need for probiotic strain (LP-B001) led to the use of the FEN DI bioreactor (Henan Fendi, China, Model Number: S212-20L, Rotating Speed of Stirring: 0-450rpm, Reaction Flask Volume: 8 L, stirring diameter of axle is 12 mm) with V_t_: 20 L and V_w_:2 L. The inoculum was expanded by 3 sequential transit of the LP- BL0111 strain in MRS culture within 48 h, isolated in optimum conditions at 27 °C. The composition of the cultivation of microorganisms in the reactor for the MRS culture medium is as follows; per 1 L of water: (Meat extract 8 g; Pepton from casein 15 g; Yeast extract 4 g; Sodium citrate 6 g, C_6_H_8_O_7_.2H_3_N 2 g). Yeast and Meat extract, Soybean and mixture of dextrose and water were sterilized (120 °C, 15 min) and maintained in 55 °C (Mirsalami and Mirsalami 2024c). After draining the bioreactor and washing the tank with distilled water, it was time for TSB culture medium (Soybean meal 3 g, Nacl 5 g, K_2_HPO_4_ 3.1g, Anhydrous Dextrose Powder 3.2 g).

### Spray-coating of MRS, TSB, and Baird parker

Before the spray-drying procedure, 255 g of the medium liquid (contains LP-B001 powder in the MRS medium) was blended with 320 g of anti-bacterial combination, cooled to 35 °C. The combination was blended for 40 min at 28 °C in 2 L continuous stirred-tank reactor (CSTR) (Parr, Illinois USA, Model Number: 4520). Eventually, such combinations were exposed to spray-drying. This process was performed for TSB and Baird parker medium. The spray drying process was initially as follows; The inlet air temperature was preheated to 180 °C, while the outlet temperature was maintained at 70 °C to minimize thermal stress on the probiotics. The probiotic suspension was then atomized using a two-fluid nozzle to create fine droplets, targeting a consistent droplet size of approximately 50 µm. The feed rate was carefully optimized to ensure a uniform drying process throughout. The dried powder was collected immediately after the spray drying process and stored in vacuum-sealed containers to prevent moisture absorption. Viability assays were performed immediately after drying and subsequently after storage at both 25 °C and 4 °C over a period of two month to assess the stability of the probiotics. The viability of the probiotics was evaluated using selective media to enumerate colony-forming units (CFUs). Additionally, scanning electron microscopy (SEM) was employed to analyze the morphology of the dried particles, providing insights into the structural integrity of the formulations.

### Effect of acidification and basification of culture medium

The pH of the environments mentioned so far was between 6.5 and 7.5. To acidify the culture medium, 0.5 ml of HNO₃ with 2 g of agar was added and finally placed on the flame for 75 s. Due to the presence of acid, the boiling point occurred at a higher temperature. Place in the autoclave (temperature 121 ℃) for 15 min. After cooling, the culture liquid was poured onto the plates and waited for the MRS and TSB medium to form on the plates. To basify the culture medium, 0.2 ml of KoH was added along with 2 g of agar.

### Dilution

Bacterial culture on basal medium was performed once directly with the primary bacterium itself and once using dilution to determine the difference between the two methods. First, the bacteria were cultured on the base medium with the help of an inoculation ring and placed in an incubator at 37 °C. Subsequently, diluted bacteria were cultured. 1 cc of physiological serum was poured into 5 tubes with 150 cc of distilled water (autoclave) and 0.45 g of bacteria was removed from the Petri dish with an inoculation loop and dissolved in the tubes containing distilled water (Bove et al. 2012; Mirsalami et al. 2024a). Then, with the help of a pipette (100 ml serology pipette), the liquid containing bacteria was removed from the tube and poured into the first tube containing physiological serum and again with the help of a pipette the same concentration was transferred to the second tube and from the second tube to the third tube and then to the fourth tube. The concentration of pipes is 1⁄100, 1⁄1000, 1⁄10.000, 1⁄100.000, 1⁄1.000.000, respectively, from a concentration of 1⁄100,000 that is diluted, 250 cc was collected and cultured and finally incubated for five minutes.

### Gram-negative bacteria detection by gram staining method

In order to be able to detect whether *Lactobacillus bacterium* itself has grown in environments or is itself a pathogenic and to detect gram-negative bacteria, Gram staining method was used. To diagnose *Lactobacillus*, a microscopic slide was removed, and a drop of physiological saline (sodium chloride solution) was poured on it, then 0.2 g of the LP bacterium (drive out from all three types of culture medium, MRS, TSB, Baird parker, respectively) was removed with an inoculation loop and dissolved with physiological saline. The liquid culture was placed on the flame for 4.5 min to dry. The slide was then placed on the paint tank, the sample was stained with Crystal violet 1% (reagent 1) for 30 s and the sample was washed with 573 ml of water (Luo et al. 2021). Following the Gram staining method, the microbial layer was covered with Lugol's iodine (reagent 2) and placed on the flame for 30 s according to reagent 1, and the sample was washed again with water. In the next step, the sample was decolorized with phenolphthalein (reagent 3) for 10–20 s and the previous steps were repeated. Finally, the sample was stained with Fuchsin (reagent 4) for 35 s and washed with distilled water. The bacterium is now easily visible under a microscope.

### Animal subjects

This study utilized a specific breed, Ross 308 broiler chickens, sourced from Khosravi Poultry Hatchery, to ensure consistency in genetic background and growth performance. A total of 280 chickens were employed, with an average age of three days and an initial body weight of 150–250 g. The chickens were housed in a controlled environment within a climate-controlled poultry house. Each group was allocated six square meters of space to ensure adequate movement and welfare. Environmental conditions, including temperature, humidity, and ventilation, were meticulously regulated throughout the experiment to maintain optimal growth conditions. Temperature was maintained at 25–28 °C during the initial phases of growth, gradually adjusted as birds matured. Humidity levels were kept between 40 and 60%, and ventilation was ensured through forced-air systems. Chickens were provided with a standard commercial diet formulated to meet the nutritional requirements established by the National Research Council (NRC) for broiler chickens. Additionally, special care was taken to monitor the health of the birds daily, with any signs of illness or distress promptly addressed. All animal husbandry practices adhered to the guidelines set forth by the Institutional Animal Care and Use Committee (IACUC), ensuring the welfare of the animals throughout the study.

### Administration of probiotic

*Lactobacillus plantarum* was administered to the broiler chickens via feed. The probiotic was incorporated into the feed at a concentration of 1 × 10^8 CFU/g of feed to ensure effective colonization of the intestinal microbiome. The duration of the probiotic treatment spanned 21 days, beginning at day 7 post-hatch, coinciding with a critical developmental phase for gut health. Chickens in the control group received a placebo treatment, consisting of the same feed without the probiotic, to account for any potential environmental or handling effects. Feeding was conducted ad libitum, and the probiotic was mixed thoroughly to ensure uniform distribution throughout the feed. Regular assessments were made to confirm that all birds were consuming the feed adequately and to monitor any adverse reactions to the probiotic administration.

### Control measures

To ensure the integrity of the experimental design, appropriate control measures were implemented throughout the study. A control group of broiler chickens was maintained without exposure to *Lactobacillus plantarum*, receiving a placebo treatment consisting of the same diet without the probiotic addition. This group served as a baseline for comparison to evaluate the effects of the probiotic on growth performance, meat quality, and gut health. In addition, all chickens were kept under identical housing and dietary conditions to minimize variability. Importantly, all antibiotic treatments were withheld during the study period to assess the natural interactions between the probiotic and the intestinal microbiome without the confounding effects of antibiotic use. Throughout the experiment, regular health checks were conducted to monitor for any signs of illness or distress, ensuring that any health issues could be promptly addressed. This strict adherence to control measures allowed for a clear evaluation of the efficacy of Lactobacillus plantarum in enhancing the intestinal microbiome and improving the overall health and quality of the broiler chickens.

### Data collection methods

To evaluate the impact of *Lactobacillus plantarum* on meat quality traits, several specific parameters were measured, including taste, texture, and nutritional value.

### Meat quality assessment

Sensory Evaluation: A trained panel conducted sensory evaluations to assess taste and texture. Samples were prepared using standardized cooking methods, and panelists rated attributes such as flavor intensity, juiciness, tenderness, and overall acceptability using a 9-point hedonic scale.

### Laboratory analyses

Nutritional value was determined through laboratory analyses, including proximate composition (moisture, protein, fat, and ash content) using established AOAC methods. Additionally, color measurements were taken using a colorimeter to evaluate visual quality.

### Gut health assessment

To assess gut health, microbiome analysis was performed on fecal samples collected from the birds at the end of the trial. DNA was extracted using a standard kit, and next-generation sequencing (NGS) was employed to characterize microbial diversity and abundance.Health metrics, including body weight, feed conversion ratio, and clinical signs of gastrointestinal health, were recorded throughout the study. Any abnormalities such as diarrhea or lethargy were noted to evaluate the overall health status of the chickens.

### Pathogen detection

To evaluate the presence and quantify pathogenic microorganisms, particularly Salmonella, several detection methods were employed.

### Culture methods

Fecal samples were collected from each group of broiler chickens at predetermined intervals (weeks 2, 4, and 6 of the study) to assess pathogen load. Samples were processed using selective media, specifically XLD agar (Xylose Lysine Deoxycholate agar), which facilitates the growth of Salmonella while inhibiting the growth of non-target bacteria. The samples were incubated at 37 °C for 24 h, and colonies exhibiting typical Salmonella morphology were confirmed through biochemical tests.

### Molecular techniques

In addition to culture methods, molecular techniques were utilized to enhance detection sensitivity. Polymerase chain reaction (PCR) assays were performed on the same fecal samples to identify Salmonella DNA. Primers specific to Salmonella were used to amplify target sequences, and the presence of PCR products was confirmed through gel electrophoresis.

### Sampling protocols

Fecal samples were collected in sterile containers to prevent contamination, with a minimum sample size of 5 g per bird. Sampling was conducted once every two weeks throughout the study to monitor changes in pathogen prevalence over time.

### Growth performance metrics

To assess the growth performance and feed efficiency of the broiler chickens, key metrics were measured throughout the duration of the feeding trial, which lasted 42 days. (I) Weight Gain Measurement: The initial body weights of the chickens were recorded at the start of the trial (day 0) and subsequently measured at weekly intervals. The total weight gain for each bird was calculated by subtracting the initial weight from the final weight recorded at the end of the trial. (II) Feed Conversion Ratio (FCR): Feed efficiency was assessed using the feed conversion ratio, calculated as the total feed consumed (in grams) divided by the total weight gain (in grams) over the trial period. Feed intake was monitored daily by recording the amount of feed provided and the residual feed remaining. (III) Measurement Intervals: Weight gain and feed intake measurements were taken weekly to facilitate time-series analysis of growth performance. These metrics allowed for the determination of trends in growth rates and feed efficiency over the course of the study.

### Measurement of fecal and serum biogenic amines

BA assessments in serum and feces employed a high-performance liquid chromatography (HPLC) (LC–MS/MS) system (Agilent 1260 Infinity II Liquid Chromatography System). For serum specimens, 250 µl of serum was directly collected for evaluation. For fecal specimens, 25 mg of freeze-dried substance was mixed with 450 μl of 75% ethanol, ultrasonicated for 25 min, and then centrifuged at 12,850 rpm for 15 min at 5 °C (Huang et al. 2022). A volume of 100 μl of the supernatant was extracted for evaluation. A total of 150 μl of serum or the fecal extract was combined with 550 μl of acetonitrile containing the internal standard dhCA (120 ng/ml), followed by vortex mixing and centrifugation to acquire 550 μl of the supernatant. The supernatant was evaporated, concentrated, and reconstituted in 150 μl of methanol, with 5 μl of this solution injected for analysis. Chromatographic separation was performed on an Agilent C18 column (2.2 μm, 1.7 mm × 120 mm) utilizing water (7 mmol/L ammonium acetate solution) and acetonitrile in a gradient elution. BA metabolites were identified in multiple reaction monitoring (MRM) modes, with quantification based on a standard curve. MassHunter software (Agilent Technologies) was utilized for BA identification.

### Fecal microbiota sequencing evaluation

Fecal microbiota sequencing was performed by Bioindicator Technologies Co. (Tehran, Iran). Total DNA was isolated from specimens, and primers aimed at the V3-V4 segment of the 16S rRNA gene were utilized (Primer Sequence: F: ACTCCTAGCGGAAGCAGCC; R: GGACTATCHGGVGTATCTAVAT). Sequencing adapters were incorporated into the primers, and the target sequences were amplified via PCR. The PCR products were subsequently purified, quantified, and homogenized to create a sequencing library. This library was sequenced using the Illumina Ion Torrent S5 System to generate raw reads. Initial data filtering based on single nucleotide quality was executed using Cutadapt. Primer sequences were eliminated using Trimmomatic. Paired-end reads were assembled with PEAR, while chimera removal was conducted using UCHIME (Mirsalami and Mirsalami 2024a). The resulting high-quality reads were grouped into OTUs with a similarity threshold of 96.5% using QIIME, and taxonomic annotation was performed using a Bayesian classifier with the RDP (Ribosomal Database Project) database as a reference.

### Enzyme‐linked colorimetric immunoassay

Cytokines (P19, LDS, IL-7, and TNF-α) were quantified using commercial ELISA kits, adhering to the manufacturer’s guidelines. These kits were obtained from Thermo Fisher Scientific (Waltham, MA, USA) (Mirsalami et al. 2024b).

### Optimal time for bacterial growth

By keeping the temperature and pH constant, the time for the growth performance of the bacteria from moment zero to the moment when the maximum growth rate of the culture medium is reached was examined. MRS and TSB prepared liquid culture medium was selected for the experiment without adding agar (Baird parker medium was removed due to unseen growth) with 10 tubes. Using a pipette, 5 cc of MRS and TSB culture medium were poured into 5 separate test tubes for each, and the tubes were transferred to the incubator. Samples were examined for up to five days.

### Temperature and storage conditions of segregated chickens

During the initial week, the chickens were maintained at a temperature of approximately 30–33 °C. As the feathers developed, the ambient temperature was gradually reduced each week: 28 °C in the second week, 24 °C in the third, 21 °C in the fourth, 18 °C in the fifth, and ultimately ranging from 11 to 14 °C starting in the sixth week. At night, when the birds were less active, higher temperatures were necessary, whereas during the day, with increased activity and food consumption, the need for warmth diminished. Consequently, the temperature for adult chickens was maintained at around 14 °C at night and between 10 and 11 °C during the daytime. The body temperature of the poultry varied between 39.5 and 40.6 °C. Adequate ventilation was implemented to ensure the health and welfare of the chickens throughout the experimental period. The airflow was consistent across the facility to prevent any hot spots or stagnant areas, creating a circular air movement that ensured thorough ventilation in all sections. Each hen had a minimum of 0.28 m^2^ of indoor space and 0.85 m^2^ of outdoor space, with their living environments regularly cleaned to minimize the buildup of litter, bacterium, and pests.

### Statistical analysis

Data collected throughout the study were analyzed using appropriate statistical methods to ensure the validity of the results. All analyses were performed using SPSS version 26, R version 4.0, which provided a robust platform for data manipulation and analysis. Descriptive statistics, including means and standard deviations, were calculated for all measured variables. To compare the effects of *Lactobacillus plantarum* on growth performance metrics, meat quality traits, and pathogen prevalence, one-way analysis of variance (ANOVA) was employed. This method allowed for the evaluation of differences between the treatment groups and the control group. When significant differences were detected, post-hoc comparisons were conducted using Tukey's HSD test to identify which groups differed from one another (Bermúdez-Humarán et al. 2024). A significance level of *p* < 0.05 was established a priori to determine statistical significance for all tests. This threshold ensured that any observed effects were unlikely to have occurred by chance.

## Results

The effect of strains in live culture medium MRS, TSB, Baird Parker was processed to determine how much the L. plantarum population progressed in these three media. From 3 culture medium MRS, TSB, Baird Parker, only Baird parker culture medium contains agar and can be well formed. But for the other two medium, a certain amount of agar was added for formation in liquid medium and Gram staining detection plates. The results of how *Lactobacillus* bacteria grow in different medium, temperature, time, substrate concentration and pH was studied and evaluated, and this laboratory scale for culture and production of probiotics in powder and liquid form was injected into broiler chickens, to compare the results concretely and finally implement it for mass production with the help of industrial bioreactors.

### Effect phenotype on *L. Plantarum*

Cell-based phenotypic assay method considered in this study, greatly help to improve the primary cultivation environment, and permit to achieve wider comprehension of microorganism efficiency in MRS (or TSB) growth medium. In the present study, the Phenotype Microarrays method showed well that BL0111 can absorb the following C sources: C_6_H_12_O_6_, C_8_H_15_NO_6_, C_12_H_22_O_11_(anhydride), Xylose, Sorbitol, Mannitol, Arabinose, Sucrose, Raffinose, D-galactoside, C_2_H_2_O_4_, C_6_H_8_O_2_, Cellobiose, salicilin, C_5_H_10_O_4_, D-arabinose, Melezitose, Trehalose, maltose, α-D-glucose, D-mannose, D-gluconic acid, Glucose, Lactose, amygdalin, D-melezitose, D-cellobiose, D-glucosamine, D-fructose, dihydroxy acetone and maltotriose.

While it has been shown that lactobacilli, especially L. plantarum, are able to ferment di, tri, and tetra saccharides due to their enzymatic activity in the neutral and basic pH range but has been observed in many cases chemical processes of carbohydrates contain sugar is limited due to competitive inhibitors.

### Effects of spraying time on composing elements of *L. plantarum*

The flavor content of the LP is listed in Table S1 as a function of spray time. Over time, the taste content of L. plantarum was gently increased until it reached an utmost in 2 min. When the spraying time was increased to 130s, those consisting mainly of apricot extract and beef extract attained the water sorption capacity, and afterwards the bulk of the suspension covered on BL0111 did not further enhancement (*ρ* > 0.07). Now this behavior was measured on a larger scale for MRS, TSB and Baird parker medium.

The cultured microorganism grew significantly in the first 24 h in MRS medium and the growth rate of Bacillus bacteria during this period was 130 mg. This growth rate continued to grow significantly even in the second 24 h (the next day) and more than doubled compared to the previous day. After 2 days, the growth rate at 350 mg suddenly stopped and we did not see any more growth in the coming days in other words, it can be said that the microorganism has reached its saturation state in the growth rate, there was no noticeable change in bacterial growth or weight gain. In general, L. plantarum grows with a steep slope for up to 48 h in MRS culture medium and the growth rate remains constant over time and the passage of time has no effect on the growth of microorganisms. In the TSB medium, the situation was different, with only 30 mg of Bacillus growing after 24 h, and this growth rate was maintained for the next 5 days.

### Appearance comparison of cultured *L. plantarum*

**Fig. 1:**
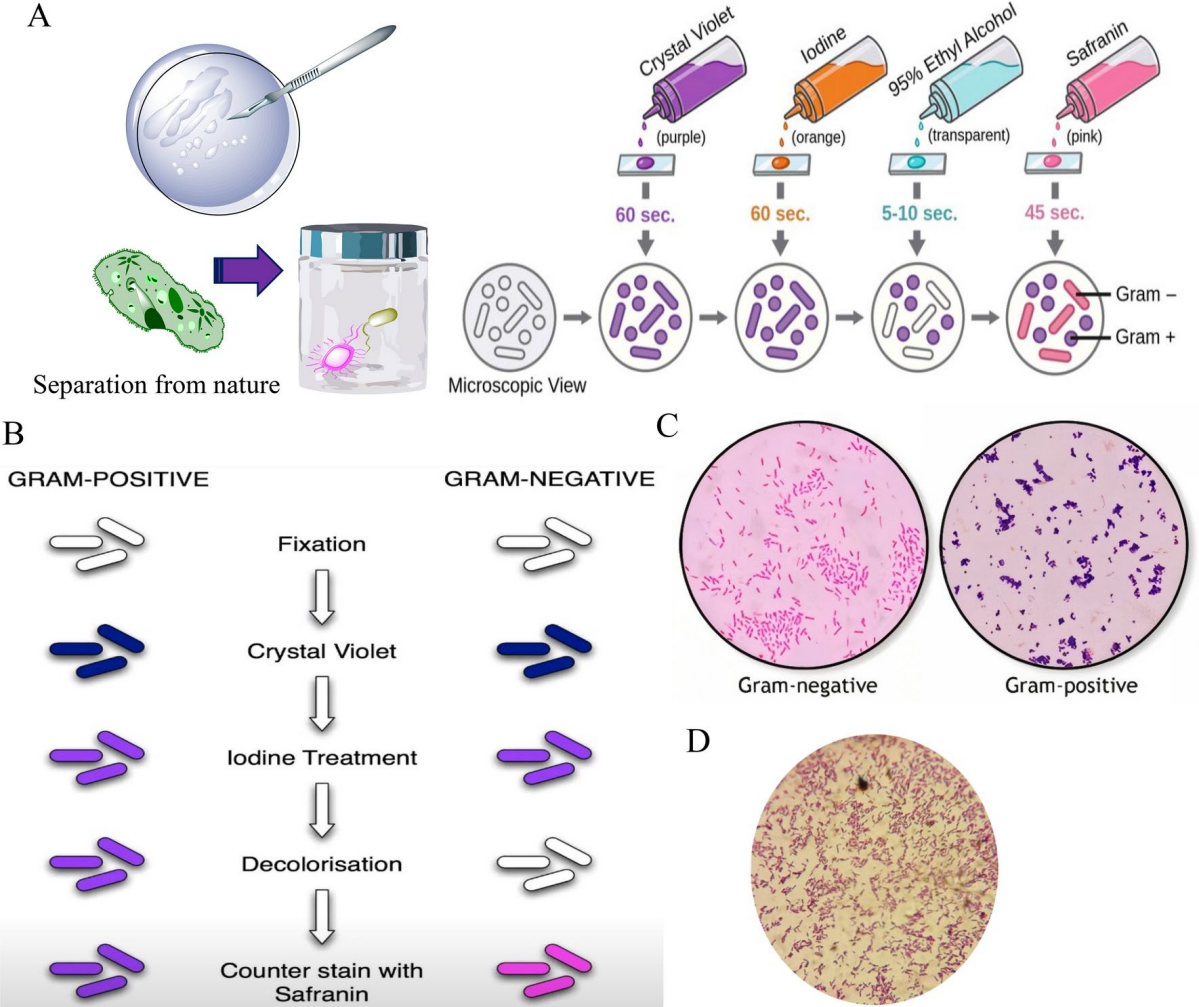
Procedure for Gram Staining; Apply crystal violet dye to the sample and let it sit for about 1 min. Rinse gently with water (**A**). Heat Fixation, Crystal Violet Staining, Iodine Treatment, Decolorization, Counterstaining Steps (**B**). Microscopy Step; Examine the slide under a microscope. Gram-positive bacteria will appear purple, while gram-negative bacteria will be pink (**C**). *Lactobacillus plantarum* under a microscope (1000x; 20:18) after 24 h of cultivation (**D**)

*L.plantarum* is purple and gram-positive. In this method, bacteria grown in neutral and alkaline media were stained with 2.10 method and can be seen under a microscope. The colonies that grew were exactly the same as L. plantarum (Fig. 1).

### Dynamic changes in the components of the culture medium

For better analysis, the role of *L.plantarum* in the growth process of the culture medium, and the composition and changes of probiotic flavoring compounds in different growth periods were compared. According to the clustering results of probiotic flavoring composites, the fugacious compounds were divided into the following categories based on the process of change in the growth and fermentation process. C_1,1_ included 7 compounds, which was varieties of meat and fish extracts. C_1,2_ included 4 compounds, were 1 aldehyde, 3 alcohols, 1 ester, 4 aldehydes. C_2,1_ included 5 compounds, which were the intermediate metabolite of L. plantarum, 1 terpene, 1 ketone, 1 aldehyde, 2 alcohols. C_2,2_ included 14 compounds, were varieties of yeat and beef extracts. Brown colors demonstrated lower than average levels of compounds and yellow colors illustrated higher levels of combination than average levels with orange.

### Growth rate in different medium

Bacterial growth in different culture media is as follows:$MRS>\text{TSB}>\text{Baird parker}$     

*L. plantarum* does not grow in Baird parker medium. *Lactobacillus* grows well in TSB medium, but the best growth belongs to MRS medium. Having nutrients such as soy, meat extract and yeast in MRS culture medium, caused unfavorable conditions (carbon deficiency, nitrogen) to grow significantly and somehow have the ability to create internal spores.

### Analysis of pH, temperature and Substrate concentration changes

In T = 36 ℃, *L.plantarum* is purple and gram-positive. By maintaining all factors unchanged and adjusting the pH towards acidity, MRS and TSB media did not exhibit substantial growth even when agar was incorporated into the plate. Bacteria experienced much better growth in the basic medium than in acidic medium and continued to grow and multiply longer, but at neutral pH, the best growth was recorded (Fig. S1).

Due to temporal changes in the growth performance of microorganisms, to achieve optimal substrate concentration and effectiveness of BL0111 in protecting probiotic survival during the culture period, 16.53 g MRS in 250 ml distilled water (in 48 h) and 9.47g TSB Dissolved in 250 ml of distilled water (within 24 h). Bacteria in MRS (after 48 h) grew almost rapidly with increasing the concentration of the substrate from 5 to 20 ml, but by increasing the concentration of the substrate, the opposite response is obtained, in fact, with increasing concentration, the substrate itself appeared as a inhibitory agent in MRS (Biabani et al. 2024). Bacteria in TSB (after 24 h) grew rapidly with increasing substrate concentration from 0 to 15 ml. Bacterial growth was no longer observed with increasing the substrate concentration from 15 ml and the growth rate remained constant (Fig. S2).

The growth conditions of the microorganism at room temperature and in the range between 20 and 42 °C were examined to ensure in which temperature range it has the best growth in solid and liquid state. After 24 h, the bacteria did not grow in the Baird parker medium, therefore, only bacteria grew in MRS and TSB medium. Bacteria do not grow well at 25 ℃ and 30 ℃ in basic and neutral environments even with dilution of the medium (Fig. S3-A). To authenticate the research findings, the experiment was expanded. First, as stated, the solid culture medium was considered for bacterial growth and the bacteria were cultured directly on the plate. The bacterial concentrations on the MRS and TSB medium were different. The bacterium had grown more in the MRS medium (Fig. S3-B) than in the TSB (Fig. S3-C). When the bacteria were dissolved in distilled water and the same concentrations were provided for both liquid culture medium and an equal amount of bacteria was cultured, the same result was obtained with solid culture medium (Zhu et al. 2022). More bacteria were observed in the test tubes containing MRS compared to TSB, ensuring that the bacteria grew better in the MRS culture medium.

According to Table 2, the broiler chickens in the group treated with *Lactobacillus plantarum* exhibited a greater increase in body weight compared to the positive control and negative control groups over a duration of 4 weeks. The weight change of chickens in three injection doses for adult chickens is shown in (Fig. S4). When comparing the food consumption among the three groups, the treatment group consumed 812 g, whereas the positive and negative control groups consumed 784 and 645 g, respectively. This indicates that the chickens in the treatment group had a stronger appetite for the probiotic containing *Lactobacillus plantarum*. The feed conversion ratio displayed varying trends throughout the entire period. In the first week, the positive control group had a higher feed conversion ratio of 1.993 compared to the treatment group's ratio of 1.581. However, in the second week, the treatment group exhibited a higher feed conversion ratio of 2.544, while the positive and negative control groups had ratios of 2.547 and 2.494, respectively. Similarly, in the third week, the treatment group maintained the highest feed conversion ratio at 3.559. Finally, in the fourth week, the negative control group had a higher feed conversion ratio than the positive control group, but both groups had lower ratios compared to the treatment group, which recorded a ratio of 4.147 kg.

**Table 2:** Effect of probiotic supplements on treatment and growth performance of broiler chickens

Parameters	Growth medium rate									SEM	*p*-value
	Sample 1			Sample 2			Sample 3				
	NC	PC	LP	NC	PC	LP	NC	PC	LP		
*FC (g)*											
Week 1	645^a^	784^b^	812^c^	645^a^	784^b^	812^c^	645^a^	784^b^	812^c^	58.1	0.021
Week 2	1.375^a^	1.275^ab^	1.951^b^	1.370^a^	1.096^cd^	1.946^b^	1.286^ab^	1.101^c^	1.092^cd^	44.7	0.003
Week 3	1.498^b^	1.412^ab^	2.312^a^	1.409^ab^	1.318^c^	1.491^b^	1.298^bc^	1.301^bc^	1.313^c^	76.5	0.025
Week 4	4.498^a^	4.012^ab^	4.512^a^	4.021^ab^	3.976^bc^	3.991^ac^	3.108^cd^	3.219^cd^	3.968^bc^	38.9	0.011
*BWG (g)*											
Week 1	391^b^	377^c^	413^a^	372^c^	289^ab^	409^a^	279^ab^	254^cd^	395^b^	11.79	0.068
Week 2	561^a^	540^b^	569^a^	491^ab^	522^cd^	545^bc^	519^cd^	449^bc^	497^ab^	6.86	0.023
Week 3	658^b^	649^b^	712^a^	614^ab^	598^bc^	707^a^	514^cd^	508^cd^	607^ab^	17.5	0.339
Week 4	1035^ab^	1016^bc^	1087^a^	1064^b^	1009^bc^	1028^ab^	839^da^	966^cd^	975^cd^	12.9	0.441
*FCRG (Kg)*											
Week 1	1.578^ab^	1.993^a^	1.581^ab^	1.591^b^	1.433^bc^	1.598^b^	1.326^de^	1.388^cd^	1.423^bc^	0.06	0.005
Week 2	2.547^a^	2.494^b^	2.544^a^	2.221^ab^	2.489^b^	2.219^ab^	1.986^cd^	1.979^cd^	2.012^bc^	0.17	0.047
Week 3	3.412^b^	3.551^a^	3.559^a^	3.054^bc^	3.179^ab^	3.187^ab^	2.814^da^	2.956^cd^	2.717^dc^	0.22	0.068
Week 4	4.098^ab^	3.975^b^	4.147^a^	3.866^bc^	3.859^bc^	4.058^ab^	3.442^dc^	3.239^ea^	3.514^cd^	0.04	0.042

*FC* Food consumption, *BWG* Body weight gain, *FCRG* Feed conversion ratio gain, *NC* Negative control, *PC* Positive control, *LPLactobacillus plantarum*, *SEM* Standard error of the mean, ^a,b,c,d,e^ Means in the same row with different superscripts differ (*p* < 0.05)

### Meat quality traits

In this study, we assessed various meat quality characteristics of broiler chickens treated with *Lactobacillus plantarum*. Key parameters evaluated included taste, texture, color, and nutritional composition.

*Sensory Evaluation*: A trained panel conducted sensory evaluations to assess taste and texture. The results indicated a significant improvement in overall flavor and tenderness in the meat from chickens receiving the probiotic treatment compared to control groups (*p* < 0.05). Quantitative measures of juiciness and chewiness were also notably enhanced, suggesting that *Lactobacillus plantarum* contributes to superior meat quality.

*Color Analysis*: Objective color measurements were taken using a colorimeter, revealing a more desirable meat color in the probiotic group, characterized by increased redness (a*) and brightness (L* values). These findings suggest that the probiotic may positively influence meat appearance, which is crucial for consumer acceptance.

*Nutritional Composition*: Comprehensive analyses of the nutritional profile showed a significant increase in protein content and essential fatty acids in the meat from the *Lactobacillus plantarum* group. Additionally, lower levels of undesirable fat were observed, indicating an improvement in the overall nutritional value of the meat.

### Microbiome analysis

The analysis of the intestinal microbiome revealed significant alterations in microbial diversity and abundance following the administration of *Lactobacillus plantarum*. Using next-generation sequencing techniques, we assessed the composition of the gut microbiota in both the probiotic-treated group and the control group. Our findings indicated a marked increase in alpha diversity metrics, including Shannon and Simpson indices, in the probiotic group compared to controls (*p* < 0.01). This enhancement in diversity suggests a more resilient and functional microbiome, which is essential for gut health and overall well-being in broiler chickens. Taxonomic analysis revealed significant shifts in microbial composition. Notably, there was an increase in beneficial genera, such as *Lactobacillus* and *Bifidobacterium*, while pathogenic genera exhibited a reduction. Bar graphs illustrate these changes, clearly depicting the relative abundance of key microbial taxa before and after treatment. Heatmaps were utilized to depict the clustering of microbiota profiles, providing a comprehensive overview of the compositional shifts in the gut microbiome. The visual aids effectively demonstrate the probiotic's role in promoting beneficial microorganisms while inhibiting the proliferation of potential pathogens. These results highlight the capacity of *Lactobacillus plantarum* to positively modulate the intestinal microbiome, fostering a healthier gut environment that may contribute to enhanced growth performance and meat quality in broiler chickens.

### Pathogen detection results

In this study, we explored the impact of *Lactobacillus plantarum* on the prevalence and quantification of pathogenic microorganisms, particularly Salmonella, in broiler chickens. This investigation is timely, given the increasing concern over food safety and antibiotic resistance in poultry production. Sampling was conducted at various intervals throughout the experiment to determine the prevalence of Salmonella in both the control and probiotic-treated groups. Remarkably, we found that *Salmonella* was present in 45% of samples from the control group, underscoring the significant challenge posed by this pathogen in conventional poultry farming. In contrast, the probiotic group demonstrated a substantial reduction in prevalence, with only 15% of samples testing positive for Salmonella. This difference was statistically significant (*p* < 0.01), indicating that the administration of *Lactobacillus plantarum* effectively mitigated the risk of Salmonella colonization in the gut of broiler chickens.

To gain deeper insights into the actual pathogen load, we employed quantitative PCR (qPCR) methods, a highly sensitive technique that allows for the precise measurement of Salmonella levels in intestinal samples. The results revealed a mean Salmonella load of 1.2 × 10^6^ CFU/g in the control group. In stark contrast, the probiotic group exhibited a significantly reduced mean load of only 3.2 × 10^4^ CFU/g. This represents a remarkable reduction of approximately 93% in pathogen levels, highlighting the potent influence of *Lactobacillus plantarum* in controlling Salmonella populations. Statistical analyses were conducted using SPSS, employing one-way ANOVA to compare data between groups. The results not only confirmed the significant reductions in Salmonella prevalence and quantification (*p* < 0.01) but also emphasized the reliability of these findings across multiple sampling points.

### Feed efficiency

In this study, we evaluated the impact of *Lactobacillus plantarum* on feed efficiency in broiler chickens, a critical factor influencing both growth performance and economic viability in poultry production. Feed efficiency, defined as the amount of feed consumed per unit of weight gain, is a key indicator of the overall health and productivity of poultry. Throughout the trial, we meticulously monitored feed intake in both the control and probiotic groups. The results indicated that the probiotic group exhibited a higher average feed intake, with chickens consuming approximately 3,500 g of feed over the study period. In contrast, the control group had a slightly lower average feed intake of 3,200 g. This increase in feed consumption in the probiotic group may be attributed to improved gut health and nutrient absorption facilitated by *Lactobacillus plantarum*. To assess the impact of feed intake on growth performance, we measured the weight gain of the chickens at the end of the trial. The probiotic group demonstrated a significant increase in weight gain, averaging 2,200 g, compared to 1,800 g in the control group. This translates to a notable improvement in overall growth performance, suggesting that the addition of *Lactobacillus plantarum* not only enhanced feed intake but also supported better weight gain. The feed conversion ratio (FCR) is a critical metric that reflects the efficiency with which animals convert feed into body mass. In our study, the FCR for the probiotic group was calculated to be 1.59, indicating that 1.59 kg of feed were required to produce 1 kg of weight gain. In contrast, the control group exhibited a higher FCR of 1.78. This represents a significant enhancement in feed efficiency in the probiotic group, with a reduction of approximately 11% in the amount of feed required for weight gain. We employed statistical analysis using SPSS, utilizing one-way ANOVA to compare the feed intake, weight gain, and FCR between the control and probiotic groups. The results demonstrated statistically significant differences in both weight gain and FCR (*p* < 0.05), reinforcing the positive impact of *Lactobacillus plantarum* on feed efficiency.

### Statistical analysis

In this study, we conducted a comprehensive statistical analysis to evaluate the significance of the findings related to the impact of *Lactobacillus plantarum* on various performance metrics in broiler chickens, including pathogen prevalence, feed efficiency, and weight gain. The statistical analyses were performed using SPSS, which facilitated rigorous testing of our hypotheses. To assess the differences between the control and probiotic-treated groups, we utilized one-way Analysis of Variance (ANOVA) for continuous variables such as weight gain, feed intake, feed conversion ratio (FCR), and pathogen quantification. This method allowed us to compare means across multiple groups while accounting for variability within the data. For post-hoc analyses, we applied Tukey's Honest Significant Difference (HSD) test to determine which specific groups differed from one another when the ANOVA indicated significant effects. This approach provided a robust framework for understanding the differences in performance metrics attributed to the treatment with *Lactobacillus plantarum*. The results of our analyses revealed statistically significant differences across various parameters. For instance, the difference in weight gain between the probiotic group (mean = 2,200 g) and the control group (mean = 1,800 g) was found to be significant with a *p*-value of 0.003, indicating a strong likelihood that the observed difference was not due to random chance. Similarly, the feed conversion ratio (FCR) demonstrated a significant difference, with the probiotic group showing an FCR of 1.59 compared to 1.78 in the control group (*p* < 0.01). These findings underscore the efficacy of *Lactobacillus plantarum* in improving feed efficiency. In terms of pathogen detection, the reduction in Salmonella prevalence from 45% in the control group to 15% in the probiotic group was statistically significant (*p* < 0.01). Furthermore, the quantification of Salmonella showed a mean difference of 1.17 logarithmic CFU/g between the groups, which was also significant (p < 0.001). In addition to *p*-values, we calculated 95% confidence intervals (CIs) for key metrics to provide a range within which we can be confident that the true population parameter lies. For instance, the 95% CI for the difference in weight gain was [300 g, 600 g], indicating a reliable estimate of the expected improvement when using *Lactobacillus plantarum*.

### Variations in bile acid concentrations across WPLP and NPLP broilers

Bile acid (BA) profiling was performed on fecal and serum samples from 200 broilers, identifying a total of 17 bile acids: cholic acid (CA), chenodeoxycholic acid (CDCA), ursodeoxycholic acid (UDCA), lithocholic acid (LCA), deoxycholic acid (DCA), hyodeoxycholic acid (HDCA), glycocholic acid (GCA), glycine chenodeoxycholic acid (GCDCA), glycine ursodeoxycholic acid (GUDCA), glycine lithocholic acid (GLCA), glycine deoxycholic acid (GDCA), taurocholic acid (TCA), taurochenodeoxycholic acid (TCDCA), tauroursodeoxycholic acid (TUDCA), taurolithocholic acid (TLCA), taurodeoxycholic acid (TDCA), and taurohyodeoxycholic acid (THDCA). The serum level of taurohyodeoxycholic acid (THDCA) was too low to be included in the statistical analysis. Primary bile acids were defined as CA, CDCA, and their conjugated variants, while UDCA, LCA, DCA, HDCA, and their conjugated forms were classified as secondary bile acids. Unconjugated bile acids consisted of CA, CDCA, UDCA, LCA, DCA, and HDCA, with the remaining bile acids being conjugated species. 12α-hydroxylated bile acids included CA, DCA, and their conjugated counterparts. Non-12α-hydroxylated bile acids comprised CDCA, UDCA, LCA, HDCA, and their conjugated forms.

**Fig. 2:**
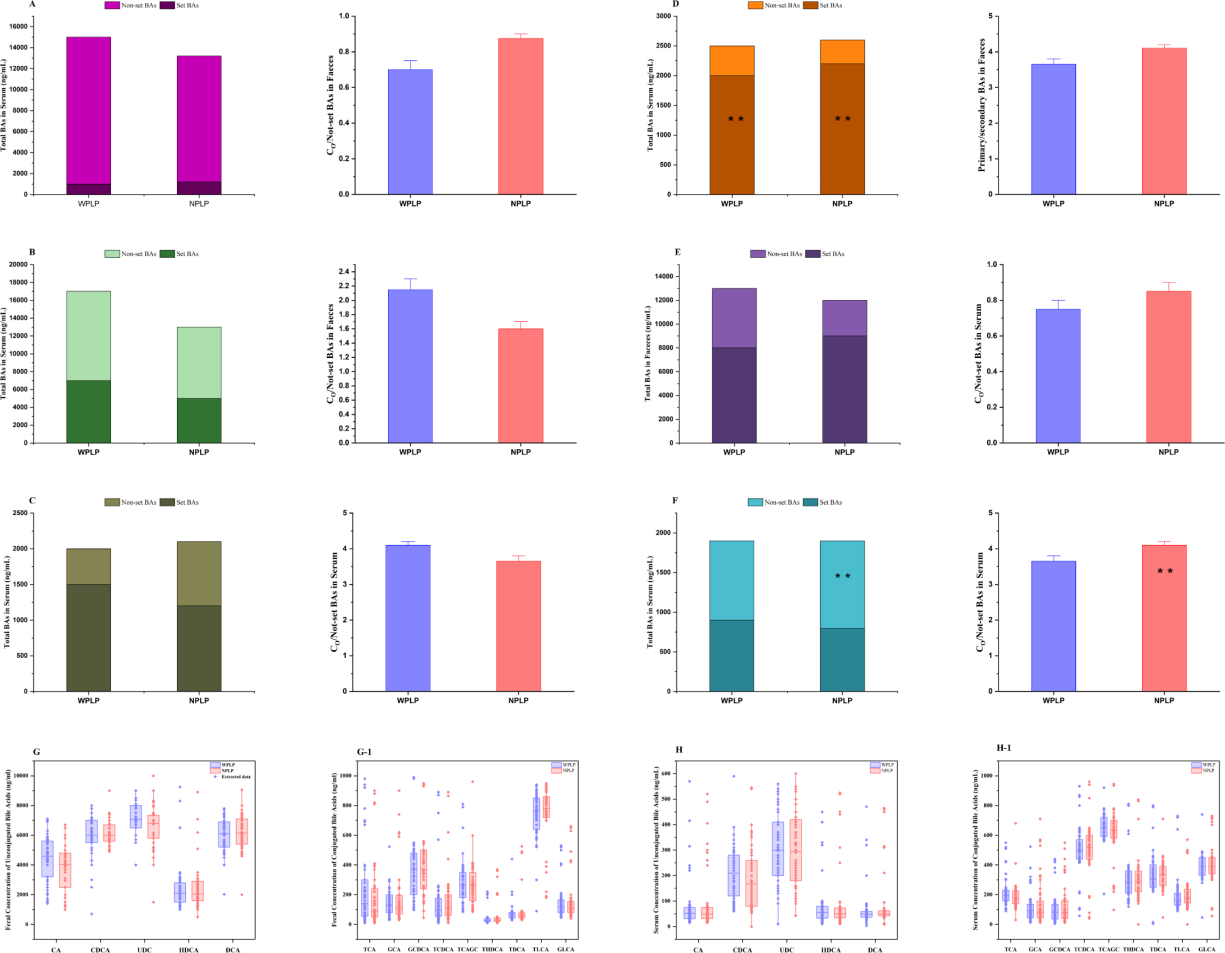
BA profile in the feces and serum of WPLP and NPLP broilers. This includes total BAs ratios of conjugated to unconjugated, and primary to secondary BAs, along with concentrations of individual BAs in feces (**A**–**C**) and serum (**D** and **E**). Ratios of 12α-OH BAs to non-12α-OH BAs in feces (**G**) and serum (**H**) are also presented. Data are expressed as mean ± SD or median (25th, 75th percentiles), based on their distribution normality. Differences in means were analyzed using an independent samples t-test, while medians were analyzed using the Wilcoxon rank sum test. Significant *p*-values are indicated (*p < 0.05, **p < 0.01, ***p < 0.01)

The total fecal bile acid (BA) levels did not differ significantly between the WPLP and NPLP broiler groups (Fig. 2A). Although not statistically significant, the ratio of conjugated to unconjugated BAs in the feces was higher in the NPLP broilers (Fig. 2A). The NPLP broilers had significantly lower levels of fecal primary BAs compared to the WPLP broilers (*p* = 0.001), resulting in a significantly lower primary to secondary BA ratio (*p* = 0.007) as well (Fig. 2B). No significant differences were observed in the fecal levels or the ratio of 12α-hydroxylated and non-12α-hydroxylated BAs (Fig. 2G). Individually, the WPLP broilers had significantly higher fecal levels of the primary BAs: cholic acid (CA) (*p* = 0.002) and chenodeoxycholic acid (CDCA) (*p* < 0.001), whereas the secondary BAs, lithocholic acid (LCA) (*p* = 0.034) and glycodeoxycholic acid (GDCA) (*p* = 0.020), were higher in the NPLP broilers (Fig. 2C). Serum total bile acid (BA) levels did not differ significantly between the groups. Interestingly, the ratio of conjugated to unconjugated BAs in the serum showed an opposite trend compared to the fecal samples. Unconjugated BA levels were significantly higher in the NPLP broilers (*p* = 0.033), resulting in a lower conjugated to unconjugated BA ratio compared to the WPLP broilers (*p* = 0.011) (Fig. 2D). Consistent with the fecal findings, the primary to secondary BA ratio in the serum was significantly lower in the NPLP broilers (*p* = 0.043) (Fig. 2E). The ratio of 12α-hydroxylated to non-12α-hydroxylated BAs was significantly higher in the NPLP group (*p* = 0.031) (Fig. 2H). Individually, serum lithocholic acid (LCA) levels were significantly elevated in the NPLP group (*p* = 0.006), while taurocholic acid (TCA) levels were higher in the WPLP broilers (*p* = 0.008) (Fig. 2F).

### Maturation and inflammation-related cytokines in the WPLP and NPLP broilers

**Fig. 3:**
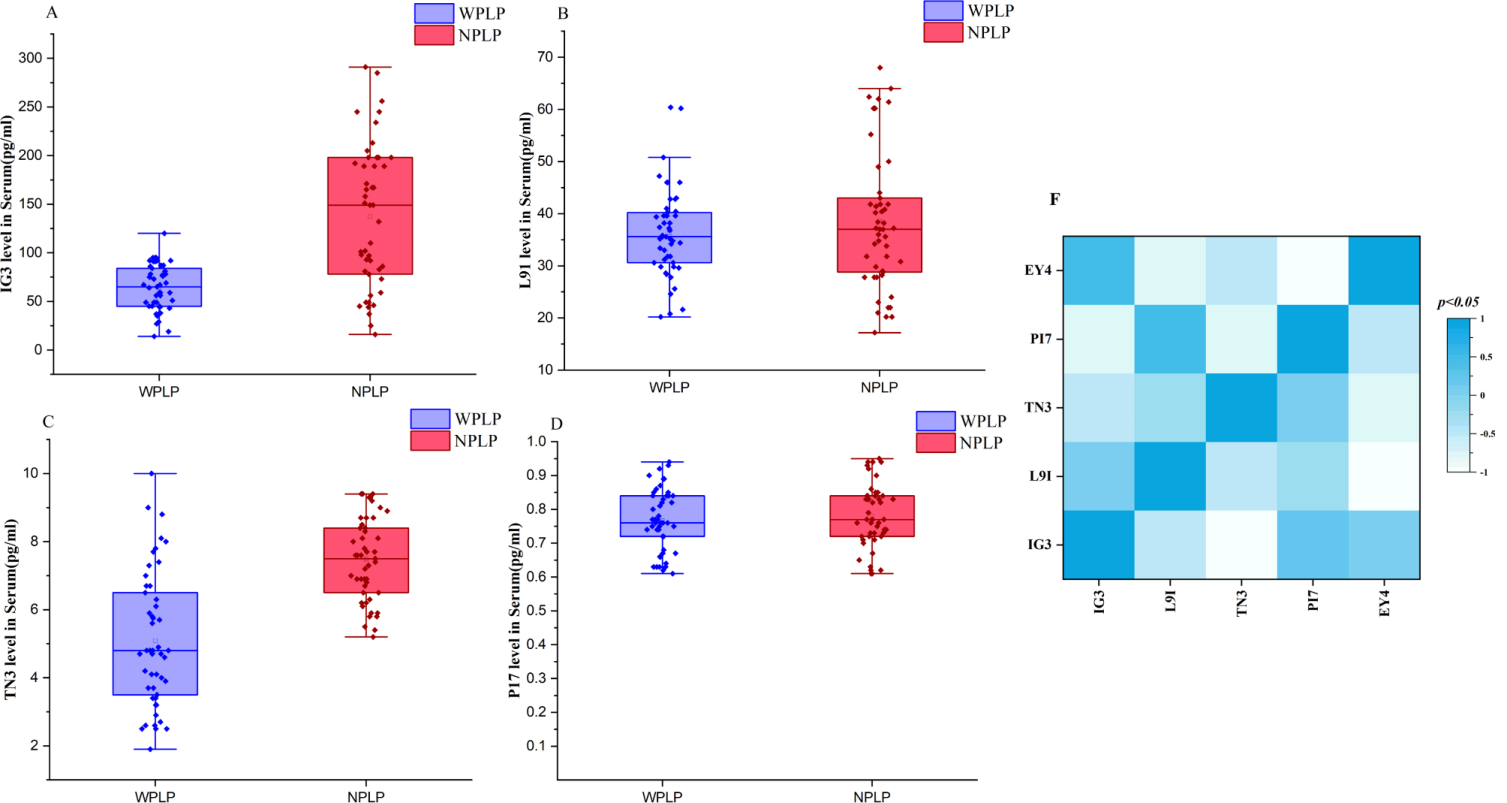
Circulating cytokine concentrations in WPLP and NPLP broilers. Enzyme-linked immunosorbent assay (ELISA) was employed to quantify the levels of p21 (**A**), lipopolysaccharide (LPS) (**B**), interleukin-6 (IL-6) (**C**), and tumor necrosis factor-alpha (TNF-α) (**D**) in the blood serum. The data is displayed as median values with the 25th and 75^th^ percentiles. Statistically significant *p*-values are denoted (*p < 0.05, **p < 0.01). (**E**) Correlational assessment among the four cytokines (*p < 0.05)

As organisms mature, their cells often release various signaling molecules, known as cytokines, which reflect the body's changes in weight and inflammatory status. One characteristic feature observed in adulthood is a "Leaky gut" in the intestinal barrier, which is associated with the aging-related processes of inflammation and immune senescence. Given the crucial role of the gastrointestinal tract in the synthesis and metabolism of bile acids (BA) by the gut microbiota, the researchers investigated whether alterations in BA profiles and gut microbiome during growth contribute to this intestinal barrier leakage and participate in the senescence process. To assess this, the researchers measured the levels of p21, a recognized marker of senescence, and serum lipopolysaccharide (LPS) levels, which are indicative of intestinal permeability. Additionally, they analyzed the levels of two key inflammatory factors, IL-6 and TNF-α. The serum levels of these four biomarkers were quantified using ELISA assays in both study groups. The results, as shown in Fig. 3, demonstrated that the levels of p21 (*p* = 0.008), LPS (*p* = 0.009), IL-6 (*p* = 0.003), and TNF-α (*p* = 0.019) were significantly higher in the NPLP (non-perforated left proximal) broiler group. These findings suggest an impaired intestinal barrier function and increased systemic chronic inflammation in the NPLP broilers, leading to a more fragile physiological state. Correlation analysis (Fig. 3F) revealed significant associations among these four cytokines, indicating a potential interplay between cellular aging, impaired intestinal barrier function, and the inflammatory response.

### Dissimilarities in gut microbial composition of WPLP and NPLP broiler groups

**Fig. 4:**
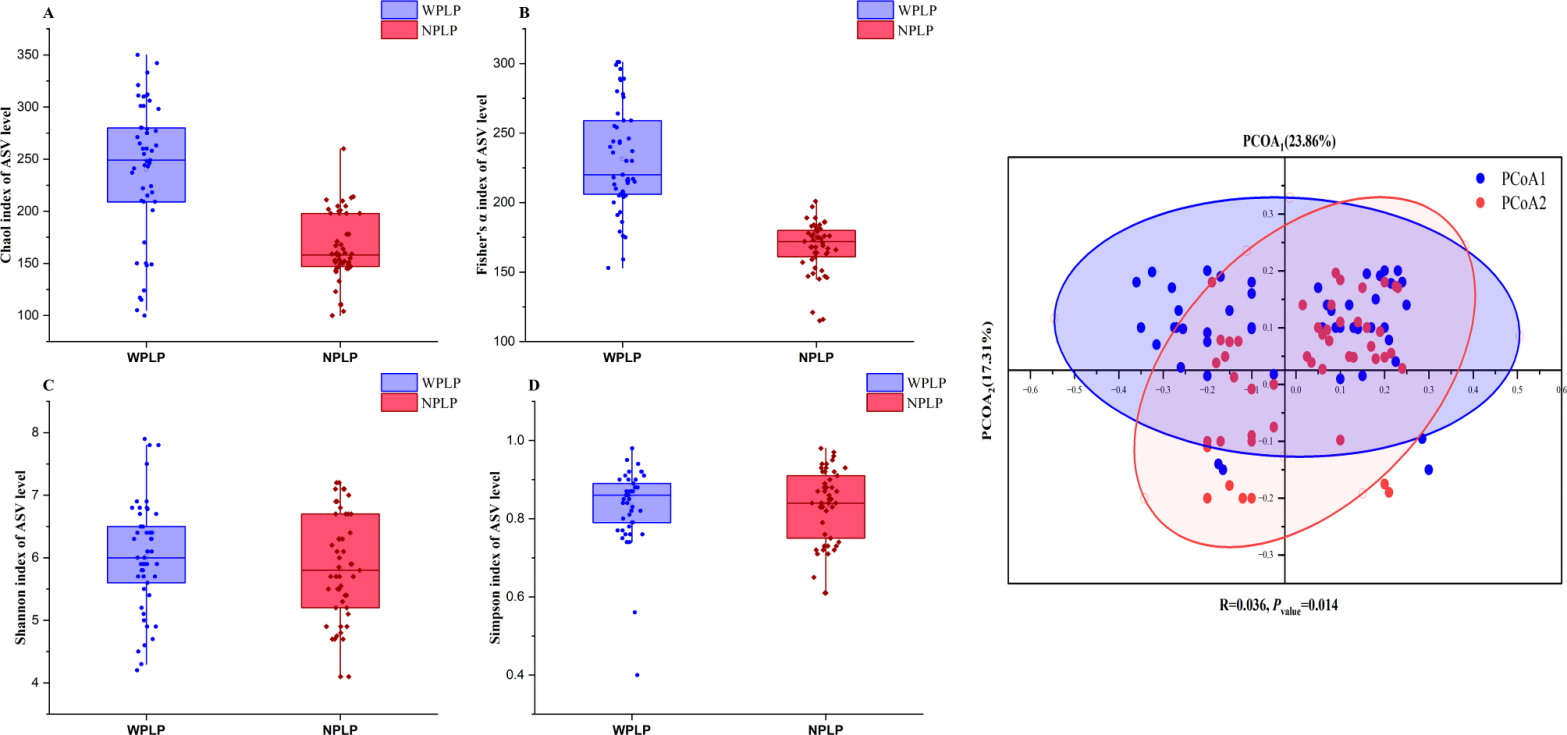
Gut microbial community profiling in the two cohorts. Species richness was evaluated at the operational taxonomic unit (OTU) level using the Chao1 index (**A**) and ACE index (**B**), while species diversity was assessed through the Shannon index (**C**) and Simpson index (**D**). Principal coordinate analysis (**E**) was performed based on the binary Jaccard distance metric. Statistically significant differences are denoted (*p < 0.05)

The research team set out to investigate the distinctions in gut microbial communities between two broiler chicken groups—the WPLP and the NPLP cohorts. Additionally, the study aimed to explore the associations between the gut microbiota and bile acid (BA) composition in these birds. To achieve this, the investigators collected and analyzed 40 fecal samples from each group, performing gut microbiome sequencing. This process generated a total of 11,244,536 paired-end reads, which, after quality control and assembly, resulted in 11,213,498 clean reads. The Usearch software was then used to cluster these reads into 469 operational taxonomic units (OTUs), with 441 OTUs shared between the two groups, 27 unique to the NPLP broilers, and 3 unique to the WPLP. Subsequent analysis of these OTUs involved evaluating alpha and beta diversity metrics to examine variations in microbial richness and community composition. As depicted in Fig. 4, the Chao1 index (Fig. 4A) and ACE index (Fig. 4B), which are indicators of species richness, were significantly higher in the WPLP group compared to the NPLP. In contrast, the Shannon index (Fig. 4C) and Simpson index (Fig. 4D), which measure species diversity, did not exhibit notable differences between the groups. This suggests that the gut microbiota of WPLP broilers harbors greater species richness, without significant disparities in species diversity, relative to the NPLP cohort. Furthermore, the principal coordinate analysis (PCoA) based on the Binary-Jaccard distance (Fig. 4E) revealed a distinct separation in the microbiota composition between the two groups (*p* < 0.05), indicating a significant difference in their overall microbial community structures.

**Fig. 5:**
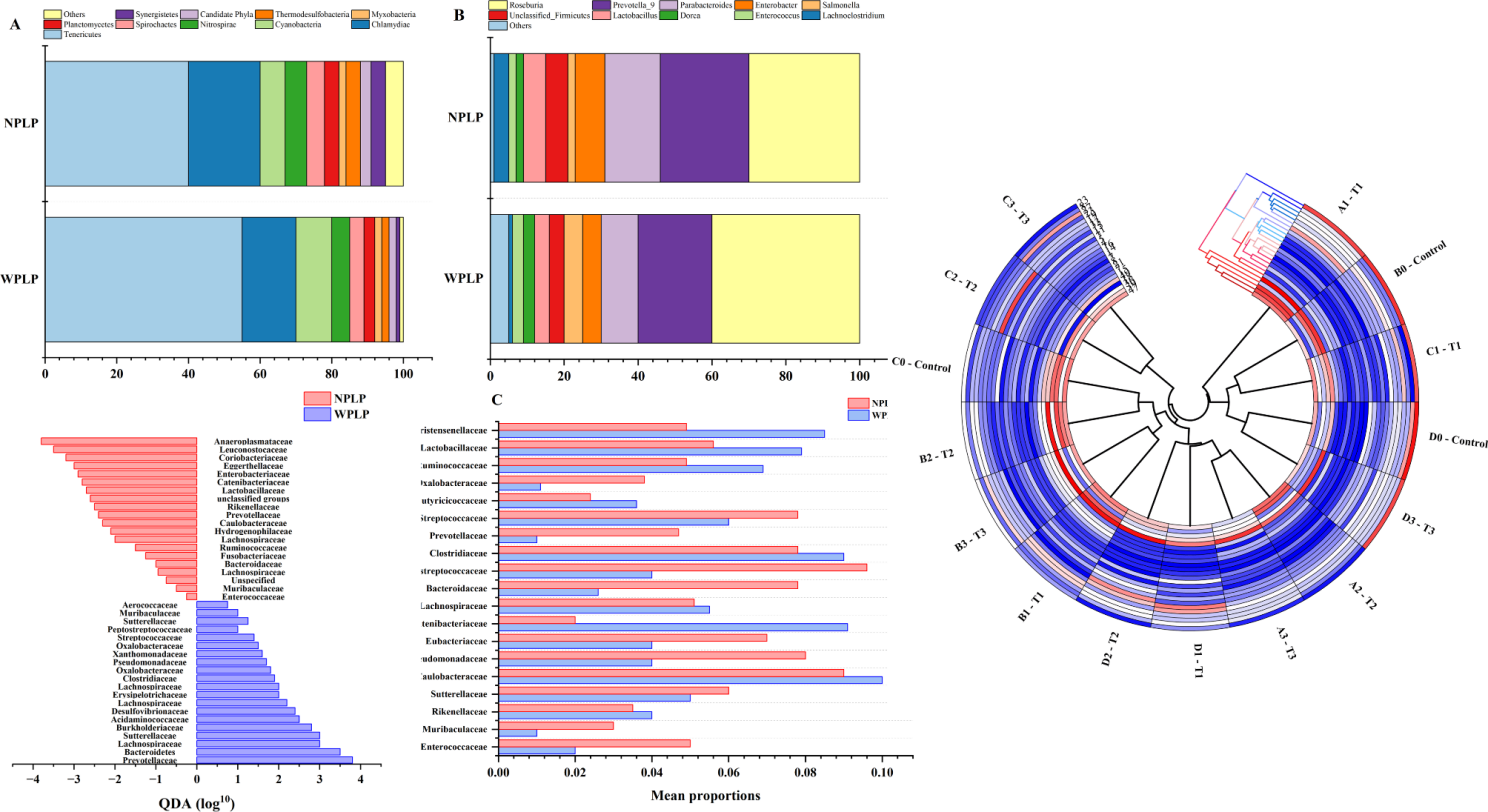
Gut microbial composition analysis. Bar plots illustrate the top 10 most abundant species at the phylum (**A**) and genus (**B**) levels based on relative abundances. **C** STAMP analysis identifies differential flora at the genus level. **D** Cladogram from LEfSe analysis. **E** Differences in gut microbiota between the two groups were identified using LEfSe analysis, with *p* < 0.05 and LDA score threshold > 2.0

The examination of microbial composition discrepancies highlighted the microbial composition across various taxonomic tiers. Figure 5A displays the top 9 most prevalent phyla, revealing that both groups' gut microbiota were predominantly composed of *Firmicutes*, Bacteroidetes, Proteobacteria, and *Actinobacteriota*, collectively constituting over 96% of all phyla. Figure 5B showcases the top 9 genera in terms of relative abundance. We then conducted a differential analysis of all gut microbial taxa at both phylum and genus levels using the Wilcoxon rank sum test in STAMP analysis. This revealed no significant differences at the phylum level. However, at the genus level, we identified 20 significantly differentially expressed gut microbiota, as depicted in Fig. 5C. Among these, *Senegalimassiliag*, *Howardella*, *Abiotrophia*, *Lactococcus*, *Oxalobacterg*, *unclassified_Lachnospiraceae*, *Pseudomonas*, *[Eubacterium]_nodatum_group*, *Holdemanella*, *Oribacterium*, Unclassified *Bacteroidales*, *Parvimonas*, *Phascolarctobacterium*, *Prevotella9*, unclassified *Muribaculaceae*, *Sutterella*, and *Brevundimonas* were relatively more abundant in WPLP-broilers.

In contrast, *Catenibacterium*, *Prevotella*, *Lactobacillus*, and *Rikenellaceae* RC_gut_group were more prevalent in NPLP-broilers. To identify distinctions in specific taxa between the WPLP and NPLP groups, we conducted a LEfSe analysis from the phylum to genus level, employing effect size measurements to highlight bacterial taxa with differing abundances between the groups. With the threshold of significance (*p* < 0.05) and LDA score > 2, a total of 37 gut microbial taxa were identified as significant, with 31 enriched in the WPLP-broilers and 7 in the NPLP-broilers. This finding aligns with the results of the STAMP analysis. Varying taxa are illustrated in Fig. 5E. Additionally, cladograms generated from the LEfSe analysis visually represent the phylogenetic distribution of these samples, from class to genus level, with the size of each circle in the cladogram indicating the abundance of specific taxa (Fig. 5D).

**Fig. 6:**
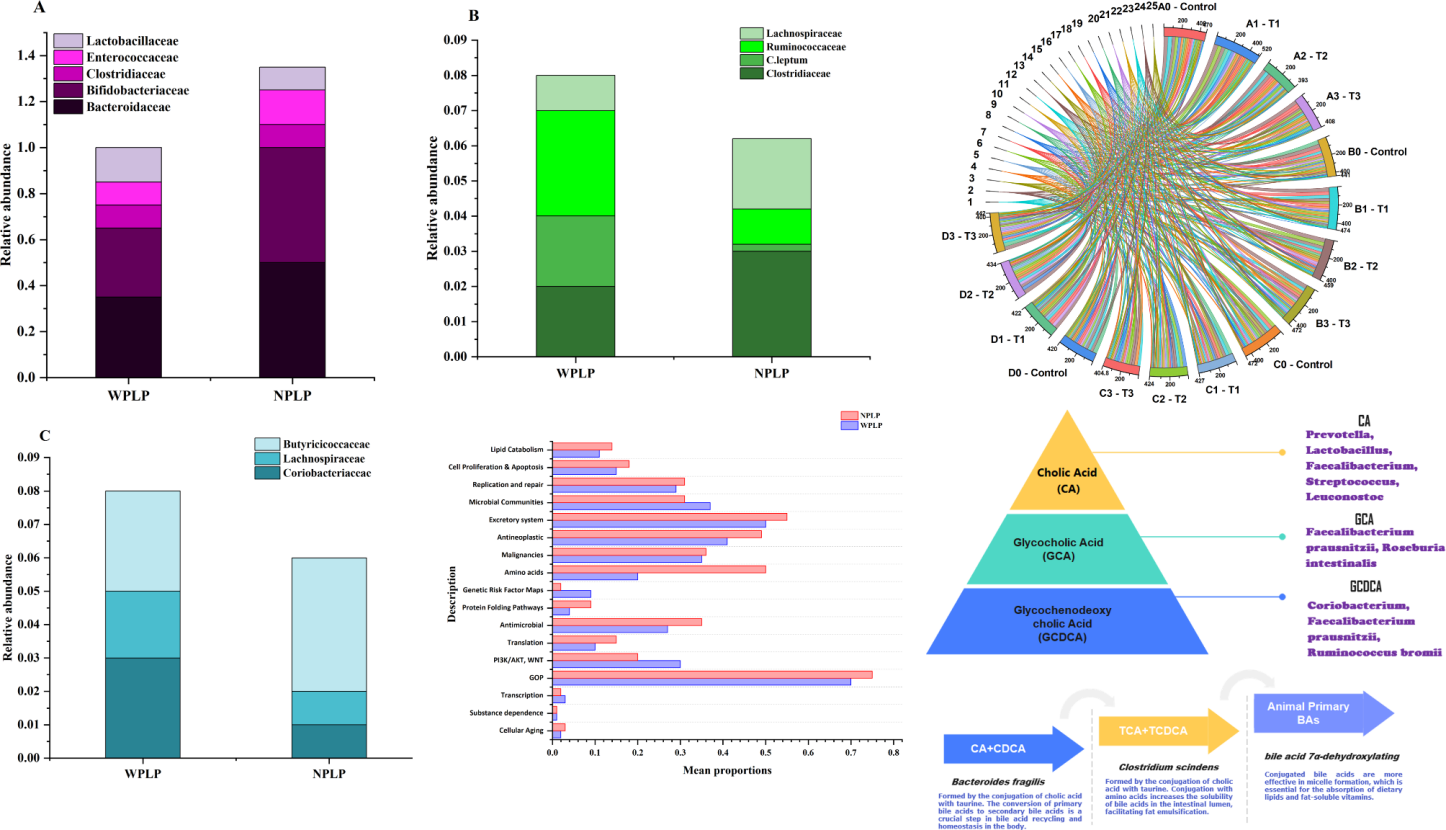
The relationship between gut microbiota and bile acid (BA) metabolism, along with anticipated functional profiles, is significant. **A** Key biological conversions of BAs by gut microbiota are highlighted. The gut microbiota composition associated with BSH (**B**), 7α-dehydroxylase (**C**), and 7α/β-HSDH (**D**) is examined. **E** Using PICRUSt 2, the functional abundance of gut microbiota was estimated, and a differential functional analysis was performed at KEGG level 2, with significance marked by *p < 0.05

The gut microbiome plays a critical role in the transformation of bile acids (BAs), with its composition and abundance being reciprocally influenced by the presence of these compounds. Around 5%-10% of the total BAs secreted end up in the colon, where they undergo microbial biotransformation before being reabsorbed or excreted in feces. As depicted in Fig. 6A, the impact of the gut microbiota on BA composition and the synthesis of secondary BAs involves three key processes: deconjugation, dehydroxylation, and oxidation-isomerization. Bile salt hydrolase (BSH) enzymes catalyze the deconjugation of BAs, leading to the formation of unconjugated forms such as cholic acid (CA) and chenodeoxycholic acid (CDCA). Subsequently, bacterial dehydroxylation enzymes convert CA and CDCA into the secondary BAs deoxycholic acid (DCA) and lithocholic acid (LCA), respectively. Furthermore, CDCA can be transformed into ursodeoxycholic acid (UDCA) by steroid dehydrogenase (HSDH), which can then be further converted to LCA through dehydroxylation.

To further investigate the connection, we analyzed the gut microbiota previously found to be associated with key enzymes involved in bile acid (BA) biotransformation, including bile salt hydrolase (BSH), 7α-dehydroxylase, and 7α/β-hydroxysteroid dehydrogenase (HSDH). Interestingly, the overall abundance of BA BSH-associated gut microbiota did not differ significantly between the groups (*p* = 0.544) (Fig. 6B). However, a closer examination revealed that the abundance of *Lactobacillus* was significantly increased in the NPLP broiler groups (*p* = 0.014), while the levels of *Bacteroides* (*p* = 0.583), *Bifidobacterium* (*p* = 0.525), Clostridium (*p* = 0.104), and Enterococcus (*p* = 0.413) did not show significant differences. Furthermore, our sequencing data identified *Clostridium_scindens* and *Clostridium_leptum* as the crucial bacterial groups responsible for BA *7α-dehydroxylation*, highlighting their pivotal role in this metabolic process. Interestingly, our analysis revealed that the abundance of Clostridium_scindens was significantly increased in the NPLP broiler group (*p* = 0.003), while the levels of *Clostridium_leptum* remained largely unchanged (*p* = 0.245). Furthermore, the overall abundance of these two bacterial groups was significantly higher (*p* = 0.005) in the NPLP broilers (Fig. 6C), which may help explain the observed differences in fecal bile acid (BA) profiles between the two groups. Additionally, *Clostridium_scindens* was found to have a BA hydroxysteroid dehydrogenase (HSDH) effect, while the other two HSDH-active bacteria, *Eggerthella* (*p* = 0.316) and *Ruminococcus_gnavus* (*p* = 0.521), showed a tendency to be elevated in the WPLP groups, although the differences were not statistically significant. Consequently, the overall abundance of these HSDH-associated bacteria did not differ significantly between the two groups (*p* = 0.872) (Fig. 6D).

### Cost analysis for economic feasibility

This study includes a detailed cost analysis for the production of *Lactobacillus plantarum* probiotics on an industrial scale, demonstrating its economic feasibility. The analysis outlines the costs associated with the raw materials, including culture media such as MRS, TSB, and Baird Parker, which collectively amount to approximately $0.50 per kilogram of finished product. Additionally, the costs of fermentation equipment and spray-drying processes, including energy consumption, have been calculated to be around $1.00 per kilogram. In comparison to alternative methods, the total production cost for our *Lactobacillus plantarum* probiotics is approximately $1.50 per kilogram. This contrasts with conventional probiotic formulations, which can range from $2.00–$3.00 per kilogram, and antibiotic treatments that often exceed $1.80 per kilogram when factoring in the costs of veterinary care and potential losses from reduced poultry health. Moreover, the economic analysis highlights that the use of *Lactobacillus plantarum* probiotics not only reduces production costs but also leads to long-term savings by improving feed efficiency and decreasing veterinary expenses. By integrating these probiotics into poultry diets, producers can achieve better growth performance and overall health in broiler chickens, making this method a cost-effective alternative to traditional practices. This comprehensive cost analysis emphasizes the practical applicability of our findings and underscores the potential for *Lactobacillus plantarum* probiotics to become a valuable tool in sustainable poultry production.

## Discussion

*Lactobacillus* serves as a favored alternative to antibiotic application in the poultry sector to mitigate the spread of antimicrobial resistance. Probiotics are beneficial microorganisms known for their antibacterial properties and their ability to promote growth. This research seeks to identify and evaluate the impact of LP species on broiler chickens. Obtain findings showed that MRS (− C(= O) − OH) without antibiotics revealed the highest fatality rate, which is consistent with the previous findings (Mirsalami and Mirsalami 2024b). Table 2 presents the effects of the LP supplement on diarrhea management and weight gain in young chickens. The enriched ingredient of daily feed tends to increase (*p* = *0.068*) the body weight gain (BWG) from the second week and considerable increment on the 14th day (*p* = *0.021*) and the throughout tryout period (*p* = 0.441) compared to the only peptone from casein and meat extract. Our outcome agreed with an investigation (Sampath et al. 2021) who intake a higher corn-soybean with LP supplementation. Correspondingly, in one study (Faramia et al. 2024) reported that supplement LP had improved the BWG of chickens. It is worth mentioning, that the supply of a nourishing diet with essential carboxyl groups and protein-forming units is fundamental for effective forage utilization (Kwak et al. 2024). The amplification of the organic acid group by the growth of microorganisms BL0111 might have been undesirable for Salmonella growth. On the one hand, food containing LP supplements arrive into the enteric tissue it might turn sugar compounds into dextrose and levulose, decrease the pH of the enteric, and progress the activities of formaldehyde, hydroxyl groups, lipids, and proteolytic enzymes to gain the length of intestinal lint, which is favorable for the uptake and breakdown of avian feed (Högberg and Lindberg 2006).

The inclusion of probiotic supplements, whether administered individually or in combination with *Lactobacillus plantarum*, led to enhanced feed intake (FI), body weight gain (BWG), and feed conversion efficiency (FCE). Significantly, the positive effects of LP (BL0001) were more pronounced in comparison to supplements containing penicillin or its analogs. Our finding was that meat and yeast extract were not very efficient, as extracted results that broilers in the TSB medium gained higher FCR and lower weight gains that were approximately similar to the outcomes the MRS medium. Chicken’ feed with the combination of probiotics remarkably accelerates growth performance and reduces FCR under stress, diarrhea, anorexia, and other status (Nam et al. 2022). The impact of LP-B0111 on growth metrics in broiler chickens facing early-stage diarrhea demonstrated that probiotic supplementation with soybean meal was equally effective as antibiotic therapy in terms of improvement of healing process and growth performance. After the challenge for 2 weeks, the BWG of broiler fed with LP (MRS) supplement (1978 ± 38.6) was considerably higher (*p* < 0.05) than the TSB (1589 ± 22.1) supplement. The FCR was able to show an acceptable mutation in the B.parker medium (*p* > 0.05) (1.33 ± 0.74) compared to the PC group (1.28 ± 0.07) and NC (1.21 ± 0.05) in the same period (after the first two weeks), demonstrator the protecting efficacy of the LP in the induction of cellular immunity in the 3 culture media tested.

The MRS cultivation medium enhances the levels of immunoglobulins A, G, and M in broiler chickens, contributing to improved growth performance and disease resistance. Additionally, MRS and TSB culture media enriched with soybean meal and D-glucose facilitate greater food nutrient absorption, support a healthy intestinal microbiome, and strengthen gut health against environmental pathogens (Abdel-Moneim et al. 2020). However, the effect of probiotic supplements on newborn chicks’ performance by differences in medium, bacteria species, choosing the right dose, nutrient type, and optimum temperature requires subsequent investigation. Thus, by ensuring optimal operating conditions and preventing pathogens that may lead to diarrhea or nutritional deficiencies in poultry during and after growth, the findings of this research can address the issues of weight loss and infectious diseases in chickens, presenting a highly promising and feasible solution (Huang et al. 2020).

In our study, SCFAs play a significant role in gut health, influencing both pathogen inhibition and microbiome stability. An in-depth analysis of butyrate's mechanisms could elucidate its multifaceted effects on gut microbiota and pathogen dynamics, making our findings more comprehensive and relevant. Butyrate, a key SCFA produced during the fermentation of dietary fibers by gut microbiota, exerts several beneficial effects on the intestinal environment. It enhances the integrity of the gut epithelium by promoting the production of tight junction proteins, which strengthen the barrier against pathogens such as Salmonella. Additionally, butyrate can modulate the immune response, fostering an environment that limits pathogen colonization while promoting beneficial microbial populations. This dual role not only helps in pathogen inhibition but also stabilizes the gut microbiome by supporting the growth of beneficial bacteria. In terms of its direct effects on Salmonella, butyrate has been shown to impact the pathogen's cell wall integrity. It can alter the expression of genes involved in cell wall synthesis and maintenance, potentially increasing the susceptibility of Salmonella to antimicrobial agents. This action, coupled with butyrate's ability to enhance the overall health of the gut environment, underscores its importance in reducing Salmonella colonization and promoting a balanced microbiome.

In summary, our research has significantly influenced several key parameters, including the selection of optimal culture media for livestock probiotic production, enhancement of weight gain, improved feed efficiency, and reduction of diarrhea and pathogenic levels. These contributions are detailed below:

*Growth Performance*: The administration of *Lactobacillus plantarum* resulted in a notable increase in average weight gain, with the probiotic group achieving a mean weight gain of 2,200 g, compared to 1,800 g in the control group. This difference was statistically significant (*p* = 0.003), indicating that the probiotic positively influences growth rates in broiler chickens.

*Feed Efficiency*: The probiotic group exhibited improved feed efficiency, as reflected by a lower feed conversion ratio (FCR) of 1.59 compared to 1.78 in the control group (*p* < 0.01). This enhancement in feed efficiency suggests that *Lactobacillus plantarum* facilitates better nutrient absorption and utilization, thereby optimizing growth performance.

*Meat Quality Characteristics*: Sensory evaluations indicated that meat from the probiotic group scored significantly higher in terms of taste and texture compared to the control group. These findings suggest that the inclusion of *Lactobacillus plantarum* not only supports animal growth but also contributes to the overall palatability of the meat, enhancing its market value.

*Pathogen Reduction*: Importantly, our results demonstrated a significant reduction in the prevalence of *Salmonella* in the intestinal samples of the probiotic group. The probiotic treatment led to a decrease from 45 to 15% in Salmonella-positive samples, underscoring the role of *Lactobacillus plantarum* in promoting food safety by mitigating the risk of pathogenic contamination.

*Overall Health Benefits*: The findings indicate that the use of *Lactobacillus plantarum* not only enhances growth performance and meat quality but also plays a crucial role in improving the gut health of broiler chickens. This probiotic reduces the reliance on antibiotics, aligning with current industry needs for sustainable and safe poultry production practices.

The competitive producing procedure of sustainable and resistant probiotics is now an essential demand of the livestock and poultry industries. Among the various strategies for incrementing the market appeal of a particular process, reducing the number of unit operations, utilizing cost-effective retention methods, and developing a sustainable growth environment, are the most promising. All influential agents were considered in the current study. Among the culture media, *L. plantarum* grew better on MRS than TSB and Baird Parker and was able to experience the best growth at 36 °C and pH = 7 (neutral). Of course, two other important factors such as substrate concentration and time were also involved, so that after 48 h in the MRS culture medium and 24 h in the TSB culture medium, no further growth was observed, and the growth rate of microorganisms reached its maximum amount. On the other hand, the growth rate of *L. plantarum* (microorganisms) in different concentrations of substrate in the two-culture media that had the highest growth (MRS, TSB) was significantly different.

The strong inhibitory activity shown by lactic acid bacteria in experiments is due to the availability of high nutrients in the growth medium of MRS, which increases the growth and metabolism of LAB so that by increasing the concentration of substrate from a certain amount, the substrate itself acts as a competitive inhibitor agent and affects the growth rate of microorganisms and ultimately reduces the growth rate of microorganisms in concentrations < 20 ml. LP-BL0111 strain displayed resistance to high temperature, alkaline culture medium, and lack of agar, and had an obvious inhibitory effect on four usual food pathogens such as diarrhea, blood in the stool, lack of absorption of nutrients in intestinal enzymes, and tumor growth. It also showed high sensitivity to penicillin. These results demonstrate that the isolated *L. plantarum* BL0111 is an ideal applicant as a dietary supplement for poultry.

## Supplementary Information

Additional file1 (DOCX 386 KB)
