# Supplementary material for: Leveraging Lactobacillus plantarum probiotics to mitigate diarrhea and Salmonella infections in broiler chickens
Source: AMB Express. 2024 Dec 18;14:137. doi: 10.1186/s13568-024-01792-3 (PMC11655822; doi:10.1186/s13568-024-01792-3)
Supplement: Supplementary file 1 — Additional file1 (DOCX 386 KB) [file 13568_2024_1792_MOESM1_ESM.docx]

**Supplementary Files**

**Title:** Leveraging *Lactobacillus plantarum* Probiotics to Mitigate Diarrhea and Salmonella Infections in Broiler Chickens

Seyed Mehrdad Mirsalami ^a,^[[1]](#footnote-1)^*^, Mahsa Mirsalami ^b^

^a^ Department of Chemical Engineering, Faculty of Engineering, Islamic Azad University Central Tehran Branch, Tehran, Iran

^b^ Faculty of Engineering and Technical Sciences, Qazvin Islamic Azad University, Qazvin, Iran

**Table S1 Content of probiotic compounds in three samples of existing medium**

| Compound | Concentrations (μg/L) | | | | | | | | | |
| --- | --- | --- | --- | --- | --- | --- | --- | --- | --- | --- |
|  | LP0 | LP6 | LP12 | LP18 | LP24 | LP30 | LP36 | LP42 | LP48 |  |
| Butanal | 9.61±45 | 5.25±58 | 2.99±32 | 8.92±02 | 6.98±28 | 3.68±1 | 2.89±93 | 4.02±11 | 1.89±41 |  |
| Tween 80 | 5.29±91 | 6.39±22 | 3.11±44 | 7.54±15 | 2.97±32 | 6.78 | 8.92±75 | 6.34±81 | 2.45±8 |  |
| 3-Octanol | 10.3±88 | 2.48 | 5.98±2 | 3.49 | 5.63±89 | 7.56±7 | 6.63 | 31.9±74 | 11.3±86 |  |
| Lithium chloride | 36.1±23 | 5.23±39 | 6.31±39 | 9.48±82 | 12.3±9 | 5.98±19 | 7.29±99 | 5.34 | 14.5±79 |  |
| 2-Ethyl-1-hexanol | 2.95 | 4.32±94 | 7.81 | 6.95±16 | 3.77±16 | 1.39±22 | 8.62±5 | 4.02±18 | 5.77 |  |
| Sodium chloride | 6.78±19 | 1.04±71 | 4.89±85 | 9.89±73 | 5.65±83 | 5.01±43 | 2.97 | 7.56±97 | 2.78±43 |  |
| Pentanal | 5.02±83 | 4.06±58 | 5.76±28 | 2.65±17 | 4.89±97 | 3.77 | 6.45±76 | 1.79 | 3.36±86 |  |
| Isopropanol | 2.98±77 | 9.69±11 | 5.68±19 | 7.15 | 7.18±12 | 5.22±03 | 8.18±18 | 9.37±08 | 6.73±79 |  |
| Isoamyl alcohol | 8.71±28 | 5.15±2 | 7.36±37 | 5.43±83 | 8.19±93 | 4.56±11 | 6.17±46 | 4.38 | 9.67±13 |  |
| 2-Pentanone | - | 7.27±87 | 15.48±4 | 2.03±03 | 5.46±78 | 3.07±08 | 8.91±28 | 3.08±78 | 18.9±48 |  |
| Hexanol | 3.56±94 | 4.09±69 | 42.01±8 | 8.34±98 | 1.12±88 | 5.72±2 | 3.19±69 | 7.11±44 | 40.6 |  |
| Oetane, 3,6-dimethyl | 61.9±28 | 25.3±47 | 4.02±89 | 7.15±71 | 3.08±93 | 47.2 | 56.2 | 4.71±52 | 5.3±77 |  |
| Propane, 1-2-methyl | 5.88±32 | 5.42±51 | 8.82±14 | 3.46±07 | 2.01 | 7.84±37 | 4.28±88 | 12.42±1 | 6.32±23 |  |
| Decane | 5.57±17 | 8.49±96 | 9.63±08 | 4.41 | - | 9.53 | 5.26±13 | 68.01±9 | 32.5±98 |  |
| Tridecane, 6-methyl | 1.91±22 | 3.09±61 | 4.48±15 | 5.67±96 | 7.15 | 2.64±96 | 3.09±76 | 12.41±2 | 2.06±03 |  |
| Nonane | 2.28±78 | 6.69±37 | 5.12 | 2.08±15 | 4.88±33 | 5.19±4 | 7.45±49 | 5.19±76 | 8.91±88 |  |
| Butanoic acid,methyl ester | 3.17±63 | 7.25±46 | 5.68±97 | 2.99±75 | 5.67±79 | 4.31 | 2.07±53 | 5.48±89 | 11.49±6 |  |
| Nonane, 3-methyl | 9.19±49 | 5.58±33 | 7.91±26 | 5.48±28 | 2.24±83 | 5.06±79 | 7.91±72 | 4.62±23 | 2.08±8 |  |
| 1,1,3,3-Tetramethylbutyl | 5.06±15 | 6.81±97 | 8.56±3 | 4.93±66 | 2.76±12 | 1.01±56 | 5.28±96 | 4.93±79 | 7.82 |  |
| Manganese sulfate | 15.7±28 | 4.32±29 | 5.07±73 | 6.49±49 | 3.744 | 1.49±75 | 6.33 | 5.79±82 | 2.93±3 |  |
| Vanillin, derivative | 2.34 | 1.09±09 | 7.94±99 | 5.58±42 | 2.82 | 3.93±48 | 17.1 | 9.39±53 | 3.13±06 |  |
| Methyl nonyl ether | 5.02±88 | 4.82±48 | 4.38±45 | 7.91±87 | 2.43±78 | 3.86±84 | 4.02±02 | 1.78±70 | 9.42±49 |  |
| Pentanedione | 7.86±37 | 6.64±07 | - | 11.48±2 | 1.78±82 | 5.69±6 | 6.79 | 8.72±96 | 3.44±56 |  |
| 2-Nonanone | 33.7±45 | 1.59±70 | 14.71±8 | 24.3±12 | 5.69±14 | 4.78±5 | 5.46±8 | 6.37±13 | 1.09 |  |
| Oxime-, methoxy-phenyl | 19.28±1 | 4.78±59 | 9.18±16 | 6.75±92 | 3.14±73 | 2.07 | 5.49±3 | 4.78±75 | 8.18±75 |  |
| 2-Heptanol | 5.3±89 | 18.8±46 | 5.73±43 | 2.48 | 9.48±22 | 5.17±73 | 9.66 | 3.08±94 | 4.17±34 |  |
| Phenethyl acetate | 6.42±67 | 5.78±37 | 9.41±95 | 5.72 | 4.14±03 | 9.43±49 | 2.83±4 | 7.04±1 | 3.49±83 |  |
| 4-Heptanol, ethyl dimethyl | 4.5±49 | 17.58±9 | 8.33±86 | 7.98±19 | 6.11±93 | 8.94±62 | 11.41±7 | 6.78 | 5.33±12 |  |
| Di-Amoniumhydrogen | 11.8±23 | 9.52±23 | 2.05±17 | 3.72±33 | 8.91±74 | 4.1 | 6.81±98 | 9.77±2 | 8.14±29 |  |
| Octyl butyrate | 8.7±91 | 5.93 | 5.81±46 | 6.09±59 | 5.82±94 | 3.9±06 | 4.89 | 6.03±07 | 4.79±45 |  |
| D-Limonene | 9.13±77 | 8.22±83 | 9.37 | 5.01±85 | 6.16±38 | 7.48±8 | 5.73±34 | 6.89±6 | 2.12±34 |  |
| Benzoic acid | 1.82±52 | 2.09±59 | 31.2±36 | 5.78±18 | 5.49±85 | 3.82±92 | 3.07±59 | 9.19 | 8.71±39 |  |
| Pentanoic acid | 5.15±41 | 18.75 | 2.98±18 | 4.98±77 | 3.16±82 | 4.55±47 | 7.09±6 | 8.14±72 | 9.86±78 |  |
| Hexanoic acid | 20.9±93 | 81.9±2 | 5.8±78 | 91.8 | 4.68±77 | 3.05±3 | 4.79 | 5.15±15 | 7.17±66 |  |
| Isooctanoic acid | 5.07±28 | 6.7±12 | 4.14 | 5.89±56 | 19.58±9 | 5.67±07 | 3.18±41 | 6.56±42 | 1.09±94 |  |
| Butyric acid | 7.59±71 | 8.15±94 | 2.19±28 | 6.47±34 | 5.52±72 | 7.17±76 | 8.19±08 | - | 9.02±46 |  |
| Acetic acid | 81.2 | 2.1±58 | 7.38 | 5.35±93 | 7.48 | 12.89 | 24.3 | 14.9±8 | 7.58±27 |  |
| Propanoic acid | 3.13±29 | 5.68±43 | 5.76±49 | 13.6±46 | 4.14±1 | 5.18±04 | 29.8±9 | 36.6±7 | 4.17 |  |
| 2-5Hexanedione | 19.77±7 | 9.08±77 | 13.13±6 | 5.86±29 | 3.1827 | 4.87±2 | 5.69±4 | 2.22 | 8.7±67 |  |
| 2-Hexen-1-ol | 2.89±51 | 15.83±2 | 63.9±79 | 8.17±85 | 3.09 | 2.99±7 | 3.41±52 | 16.8±5 | 7.91±48 |  |

Data are expressed as the mean ± standard deflection from iterates analyses (n = 3) of three repeat samples. The different lowercase letters in each row illustrate significant differences between samples (P < 0.05). The symbol “-” means not found.

^
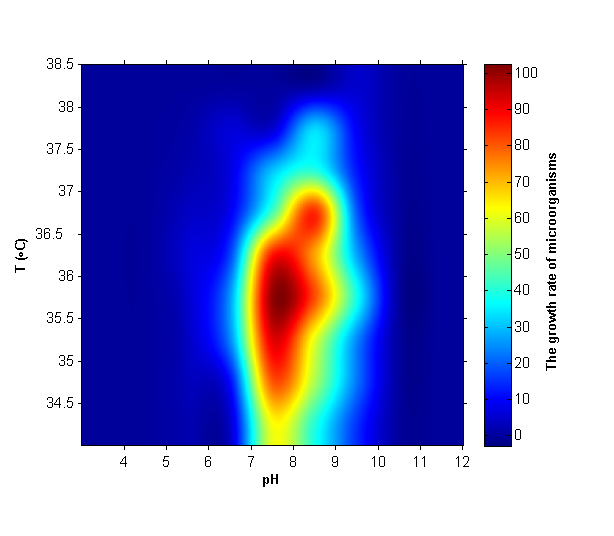
^

**Fig. S1.** Growth rate of *L. plantarum* at room temperature and neutral pH, acidic and basic


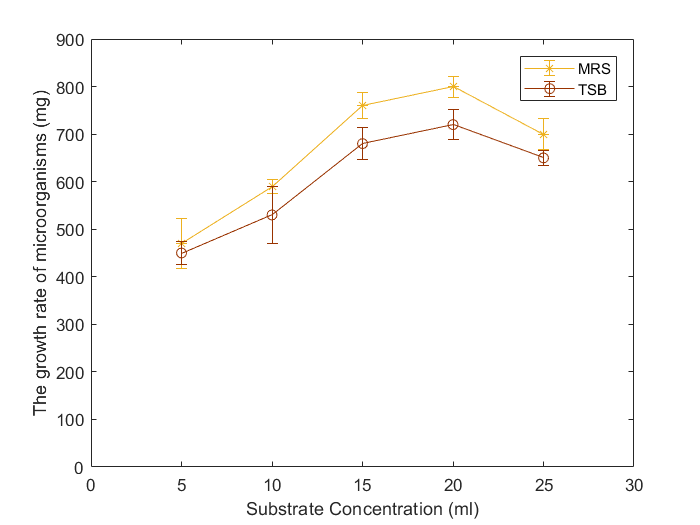


**Fig. S2.** The effect of changing the concentration of substrate (ml) in MRS, TSB medium on the growth rate of microorganisms (t = constant)


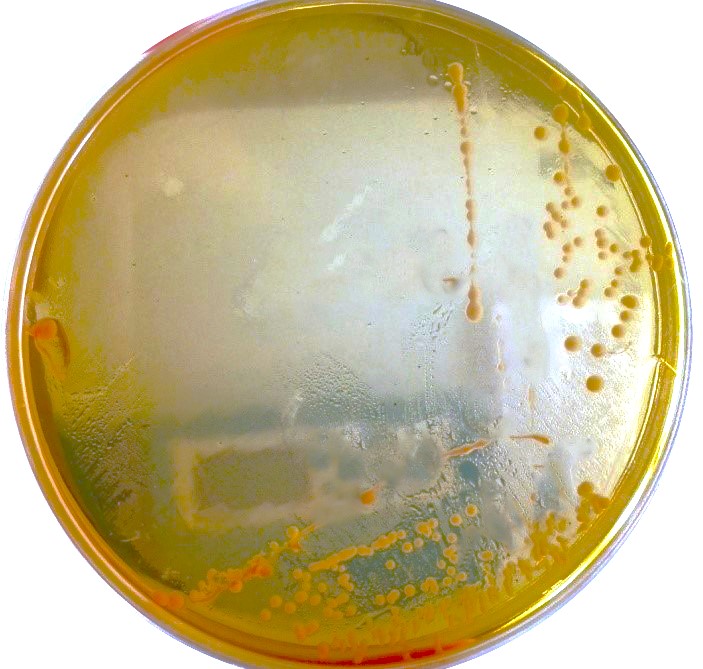

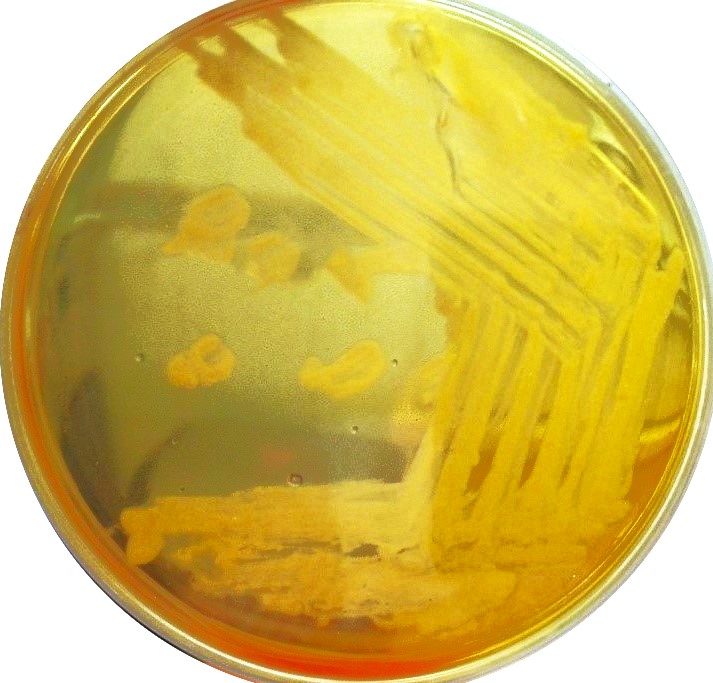


A (NotActive)

B (MRS)


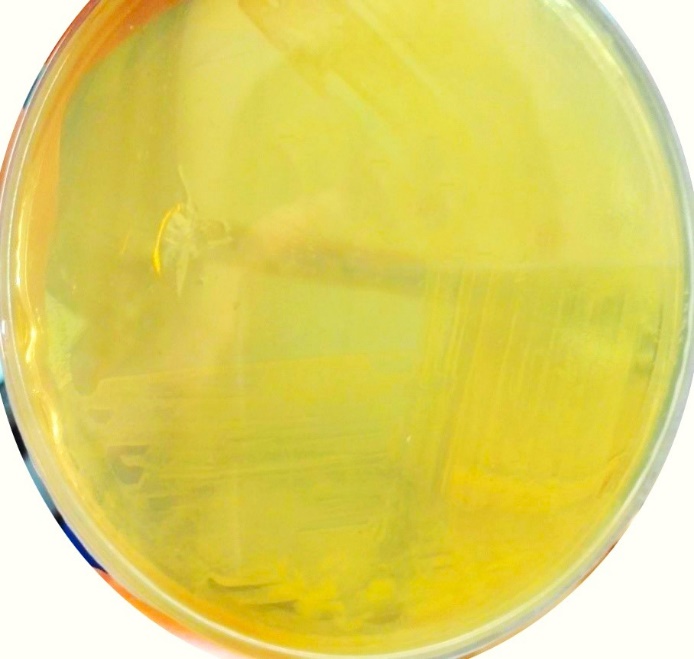


C (TSB)

**Fig. S3.** A. *Lactobacillus plantarum* strain in MRS medium at 30°C (inactivity of microorganism growth at 30°C and below). (B),(C) Bacterial growth on MRS and TSB medium, 37°C, pH = 6.5-7 respectively


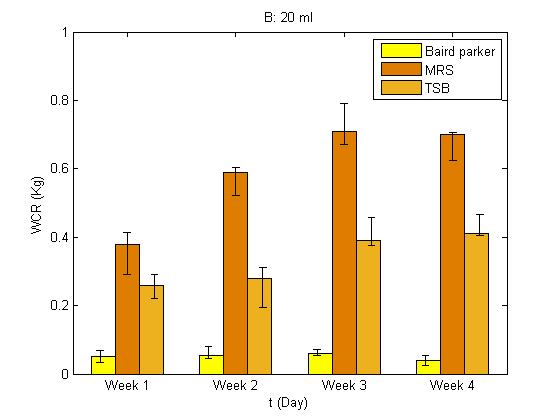

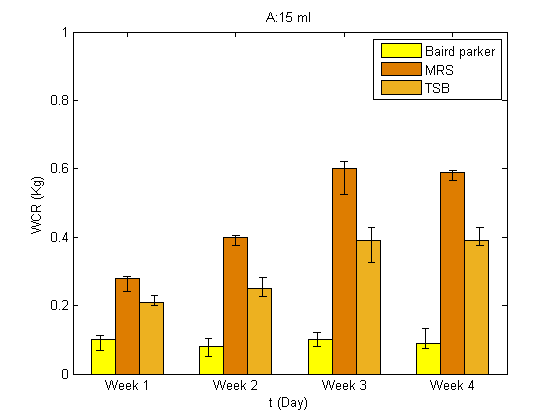


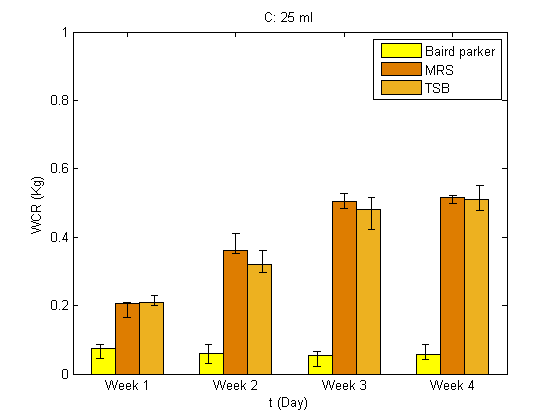


**Fig. S4.** Weight change of adult chickens after receiving 15, 20 and 25 ml within 4 weeks. A: Weight of chickens before probiotic injection respectively (2.061 Kg: Baird parker, 2.111 Kg: MRS, 2.203 Kg: TSB) -- 15 ml recipient of LPS-115 (bacteria cultured in solution), B: (1.947 Kg: Baird parker, 1979 Kg: MRS, 2.301 Kg: TSB) -- 20 ml C: (1.989 Kg: Baird parker, 2.027 Kg: MRS, 2.185 Kg: TSB) -- 25 ml (*Weight change rate: WCR)

1. * Corresponding author: Email: [m.mirsalami.eng@iauctb.ac.ir](mailto:m.mirsalami.eng@iauctb.ac.ir) (SM.M.) [↑](#footnote-ref-1)
